# 200 Years of The Haloform Reaction: Methods and Applications

**DOI:** 10.1002/chem.202403045

**Published:** 2024-11-07

**Authors:** Albert C. Rowett, David M. Heard, Priya Koria, Alice C. Dean, Stephen G. Sweeting, Alastair J. J. Lennox

**Affiliations:** ^1^ School of Chemistry University of Bristol Cantock's Close Bristol BS8 1TS United Kingdom

**Keywords:** Haloform, Halogenation, Oxidation, Ester, Carboxylic acid

## Abstract

Discovered in 1822, the haloform reaction is one of the oldest synthetic organic reactions. The haloform reaction enables the synthesis of carboxylic acids, esters or amides from methyl ketones. The reaction proceeds via exhaustive α‐halogenation and then substitution by a nucleophile to liberate a haloform. The methyl group therefore behaves as a masked leaving group. The reaction methodology has undergone several important developments in the last 200 years, transitioning from a diagnostic test of methyl ketones to a synthetically useful tool for accessing complex esters and amides. The success of the general approach has been exhibited through the use of the reaction in the synthesis of many different complex molecules in fields ranging from natural product synthesis, pharmaceuticals, agrochemicals, fragrants and flavourings. The reaction has not been extensively reviewed since 1934. Therefore, herein we provide details of the history and mechanism of the haloform reaction, as well as an overview of the developments in the methodology and a survey of examples, particularly in natural product synthesis, in which the haloform reaction has been used.

## Graphical abstract

Known for over 200 years, the haloform reaction is one of the oldest organic reactions, enabling the synthesis of carboxylic acids, esters or amides from methyl ketones via exhaustive halogenation. The reaction has not been extensively reviewed since 1934. Herein, we provide an overview of the history and mechanism of the reaction, methodology developments and a survey of applications, particularly in natural product synthesis.

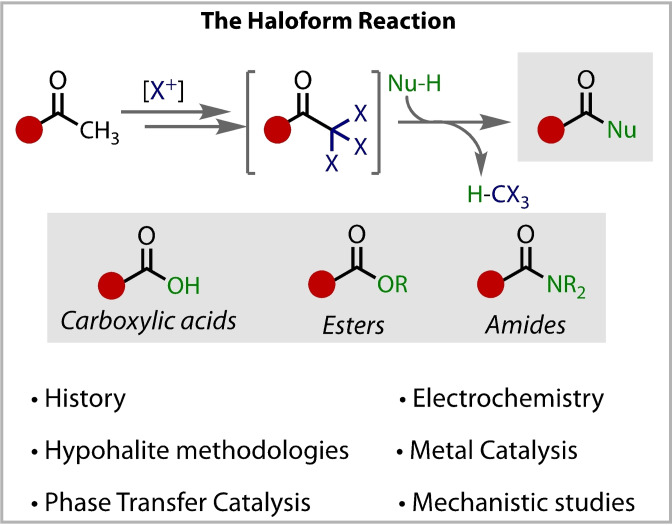

## 1 Introduction

**Figure 1:**
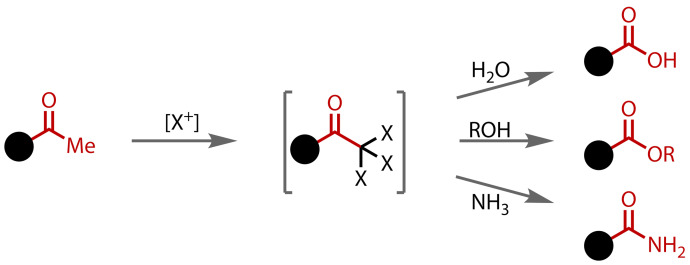
The haloform reaction can be used to transform methyl ketones to carboxylic acids, esters and amides.

The haloform reaction has traditionally been used to transform methyl ketones to carboxylic acids through the perhalogenation of the methyl group and displacement with hydroxide.[^1^, ^2^] The reaction has been further developed to directly form esters and other oxidised products, such as amides, Figure 1. While numerous general methods exist for the synthesis of these functional groups, their direct formation from ketones is more unusual. Only very limited examples exist for the conversion of ketones to carboxylic acids,[^3^, ^4^] esters[^5^, ^6^, ^7^] or amides[^8^, ^9^] via methods other than the haloform reaction. Compared to these other methods, the haloform reaction is advantageous because it is direct, uses inexpensive, readily available and safe reagents, and is amenable to scale‐up.

**Figure 2:**
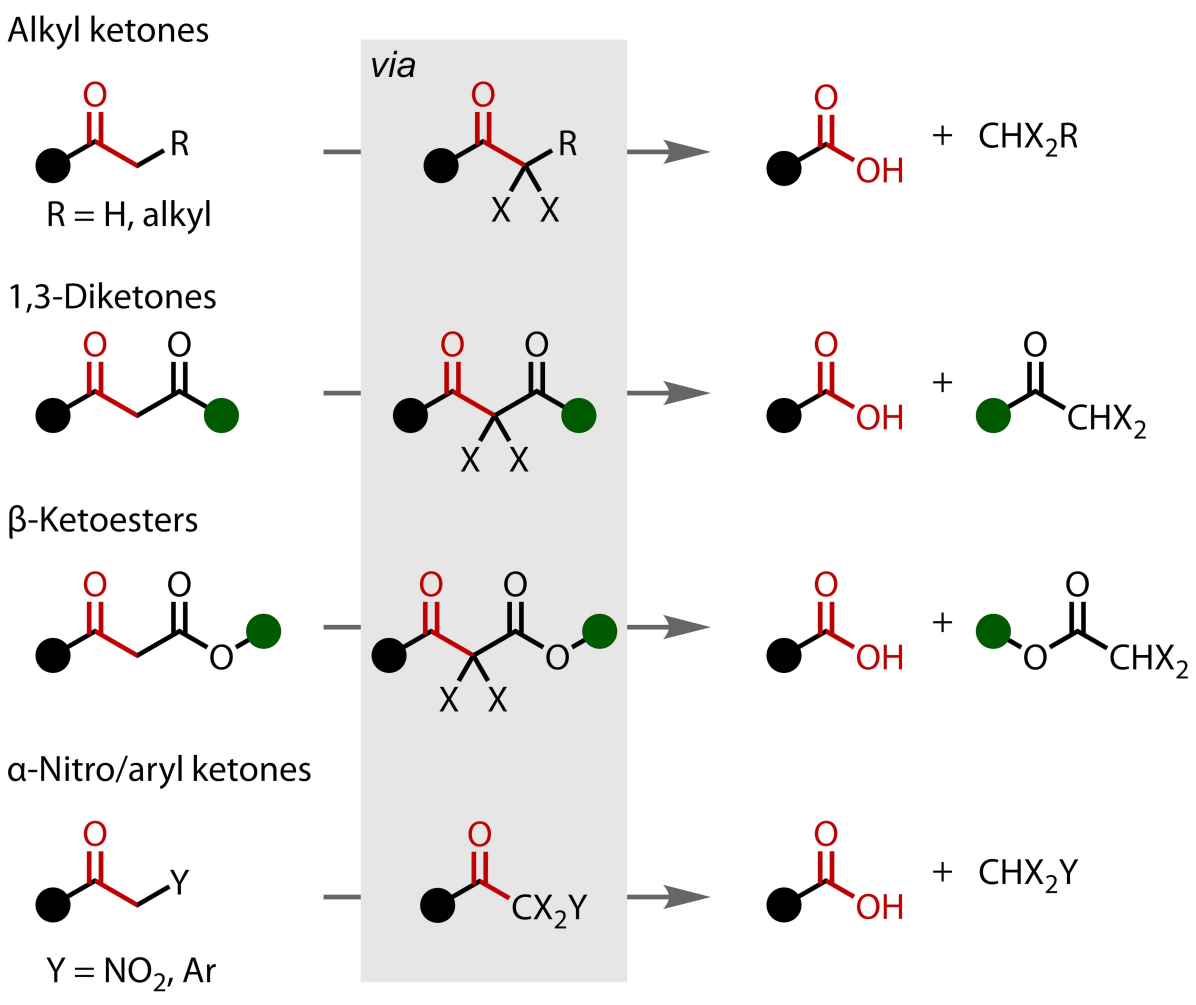
Non methyl ketone substrate classes susceptible to the haloform reaction. Ar=Aryl.

The original definition of the haloform reaction required generation of a trihalomethane (haloform) species. However, it has since been extended to include the formation of any leaving group *via* initial mono‐ or polyhalogenation of a methyl, methylene or methine unit, which is then cleaved to generate new functionality. This has extended the scope of the haloform to include several other substrate classes that react in the same way, most notably 1,3‐diketones[^10^, ^11^, ^12^, ^13^] and β‐ketoesters,^[14]^ but also α‐nitro^[15]^ and α‐aryl methyl ketones,^[16]^ Figure 2. In each case, one of the halogen atoms that would be installed has instead been replaced by an alternative, pre‐installed electron‐withdrawing group, resulting in the formation of −CX_2_Y leaving groups (where X is a halogen atom and Y is the alternative electron–withdrawing group). Although haloforms are not produced (except in the case of 1,3‐diketones, where further reaction of the dihalomethyl ketone produced does eventually yield a haloform), these examples still proceed through the intermediate formation of a α‐haloketone and therefore fit the definition.

Early insight on the haloform reaction was collated by Fuson and Bull in a comprehensive review in 1934, including detailed discussion of the discovery of the reaction and foundational history.^1^ At the time of this review, no reports on the synthesis of esters or amides from methyl ketones had been reported. However, since then, the reaction has been discussed in several textbooks.[^17^, ^18^, ^19^, ^20^, ^21^] Within these examples, Chakrabartty provided a discussion in 1978 into the mechanism and kinetics of the haloform reaction,^[20]^ which was further expanded upon by Kurti & Czako in 2005, including 4 examples of the application of the haloform reaction in total synthesis.^[21]^ Most recently in 2007, Gribble reviewed the kinetics of the reaction, as well as providing discussions around methodology development, synthetic utility, and the biological haloform reaction.^[17]^ Aside from these references in textbooks, the haloform reaction has not been reviewed since 1934.

This review, the first since Fusion and Ball's 1934 review, will briefly summarise the history and mechanism of the reaction, as well as detailing developments in reaction methodology and describing applications in organic synthesis. It will include reactions in which initial mono‐ or polyhalogenation of a methyl or methylene unit leads to formation of a haloform(−like) leaving group, which is then cleaved to generate new functionality. Developments in the scope, based on old reaction conditions will be highlighted, as well as contemporary methodology developments, organised by transformation type. An extensive overview of the use of the haloform reaction in the synthesis of different complex molecules will then be provided.

Since intermediate formation of an α‐haloketone is key for defining a “true” haloform reaction, methodologies which do not pass through such an intermediate, but which do effect the overall transformation to produce acids, esters, amides or acyl chlorides from methyl ketones will not be discussed. These include, for example, the Haller‐Bauer, Baeyer‐Villiger and Schmidt reactions,[^22^, ^23^, ^24^] as well as others that clearly proceed through alternative mechanisms.[^3^, ^25^, ^26^, ^27^] A closely–related and frequently‐used alternative to the haloform reaction is a two–step strategy, which first involves installation of a trihalomethyl ketone group that is then substituted in a separate second step. This approach, which was first reported in 1931,^[28]^ has been particularly used for the installation of ester moieties,[^29^, ^30^, ^31^, ^32^, ^33^, ^34^, ^35^] however, it has also been employed for the preparation of amides[^36^, ^37^, ^38^, ^39^, ^40^, ^41^, ^42^, ^43^, ^44^, ^45^] and carboxylic acids.[^46^, ^47^] Typically, a trichloromethyl ketone is separately prepared that is followed‐up in a second C−C bond cleavage step with methoxide to yield a methyl ester.[^29^, ^30^] However, this approach has also been used for the coupling of much larger alcohol or amine fragments.[^31^, ^32^, ^33^, ^34^, ^35^, ^44^, ^45^] Trichloromethyl ketones are sufficiently stable to be isolated and can even be carried through other synthetic steps prior to their cleavage. Further details and examples of this related, two‐step strategy, however, are considered beyond the scope of this review and are therefore not given.

### 1.1 History of the Haloform Reaction

**Figure 3:**
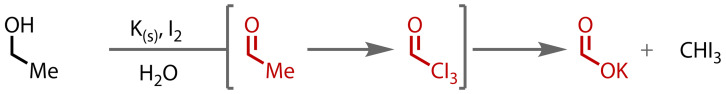
Serullas’ serendipitous discovery of the haloform reaction.

In 1822, Georges–Simon Serullas discovered that the addition of potassium to a solution of iodine in aqueous ethanol led to the formation of a yellow precipitate, which was termed a *“hydroiodide of carbon”*.^[48]^ This precipitate was actually iodoform (triiodomethane) and Serullas had, serendipitously, discovered the haloform reaction, Figure 3. Two other haloforms (so named because they produce formic acid on hydrolysis), chloroform and bromoform, were subsequently discovered by similar means in 1831 and 1834, respectively.[^49^, ^50^, ^51^, ^52^, ^53^]

Early research on the haloform reaction was focussed on the discovery of compounds that could be subjected to the reaction, with Lieben formulating a general rule in 1870: *“a positive iodoform test [iodoform production observed on addition of hypoiodite solution] is given by compounds containing the aceto (CH_3_CO−) group joined to either carbon or hydrogen, and by compounds which are oxidised under the conditions of the test to derivatives containing this structural unit”*.^[54]^

Lieben's original rule for the ‘iodoform test’ was subsequently updated by Fuson and Tullock to account for the production of iodoform in reactions from partially‐iodinated reaction intermediates, as well as to incorporate empirical evidence of the reaction's limitations: *“the test is positive for compounds which contain the grouping [sic] CH_3_CO−, CH_2_ICO−, or CHI_2_CO− when joined to a hydrogen atom or to a carbon atom which does not carry highly activated hydrogen atoms or groups which provide an excessive amount of steric hinderance. The test will, of course, be positive also for any compound which reacts with the reagent to give a derivative containing one of the requisite groupings. Conversely, compounds which contain one of the requisite groupings will give a negative test in case this grouping is destroyed by the hydrolytic action of the reagent before iodination is complete”*.^[55]^

The cleavage of pre‐formed trihalomethyl ketones by ammonia was described in reports from the 1870s, and the action of nitrogen triiodide on methyl ketones was described in 1913 as forming a mixture of iodoform and ammonia, along with an acid and an amide, with the latter forming via the reaction of *in situ* formed ammonia with the triiodomethyl ketone.[^56^, ^57^, ^58^, ^59^]

**Figure 4:**
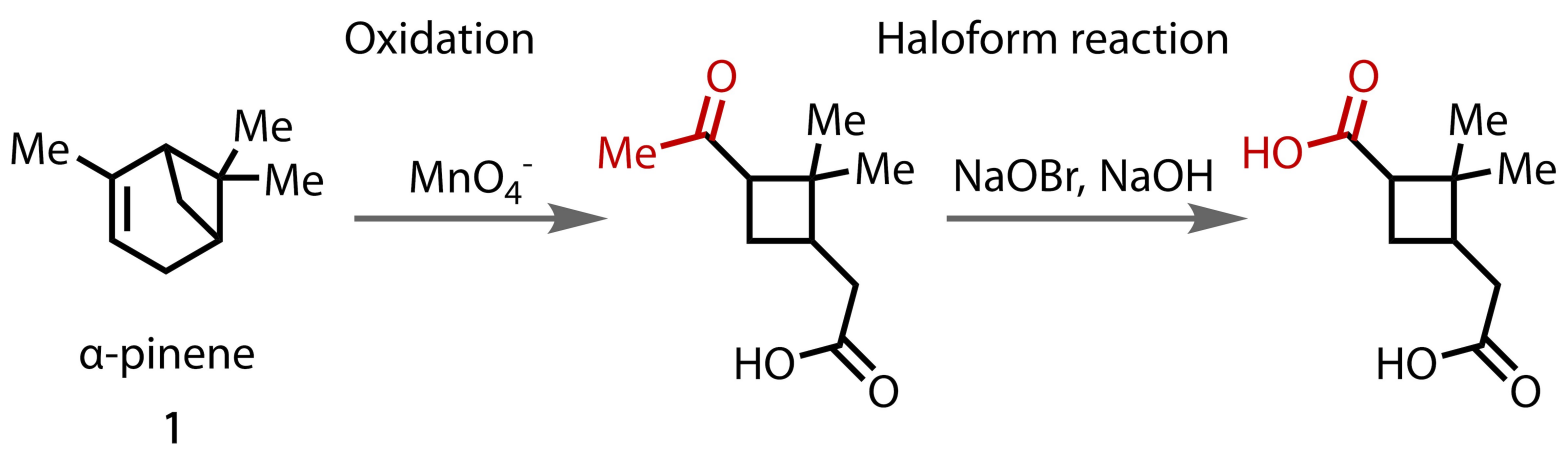
Haloform degradation contributed to the structural determination of α‐pinene, among other terpenes.

Before the introduction of modern spectroscopic techniques, the haloform reaction was a valuable tool for structure determination, owing to its high selectivity for methyl ketone oxidation. This was particularly useful in the field of terpene chemistry;^[1]^ for example, the position of the double bond in α‐pinene (**1**) was established, in part, thanks to haloform degradation, Figure 4.[^60^, ^61^] The haloform reaction has been used more recently for structure elucidation through the derivatisation of unknown isolated natural products.^[62]^

Since iodoform is an easily‐isolable, yellow solid that can be weighed and quantified, the iodoform reaction has been used for the quantitation of susceptible compounds.^[63]^ Lieben demonstrated, as early as 1870, that alcohol could be detected in aqueous solutions at concentrations as low as 500 ppm.^[54]^ Volumetric methods based on titrations of iodine with thiosulfate were also developed around this time.^[64]^

The reaction was used for the production of haloforms themselves in the decades following its discovery, including for the synthesis of isotopically pure CDCl_3_ from trichloroacetophenone in NaOD and D_2_O.^[65]^ Reports on the production of iodoform from the reaction of acetone, iodine and ammonia also date back to 1932.^[66]^ While this is no longer prevalent, the use of the haloform reaction for the synthesis of carboxylic acids and other higher oxidation state products, which did not start in earnest until the turn of the 20^th^ Century,^[1]^ has continued to the present day. This is particularly true in the field of total synthesis, where the haloform reaction is regularly called upon as a reliable method for the installation of carboxylic acids or, to an increasing degree, esters.

### 1.2 Mechanism of the Haloform Reaction

**Figure 5:**
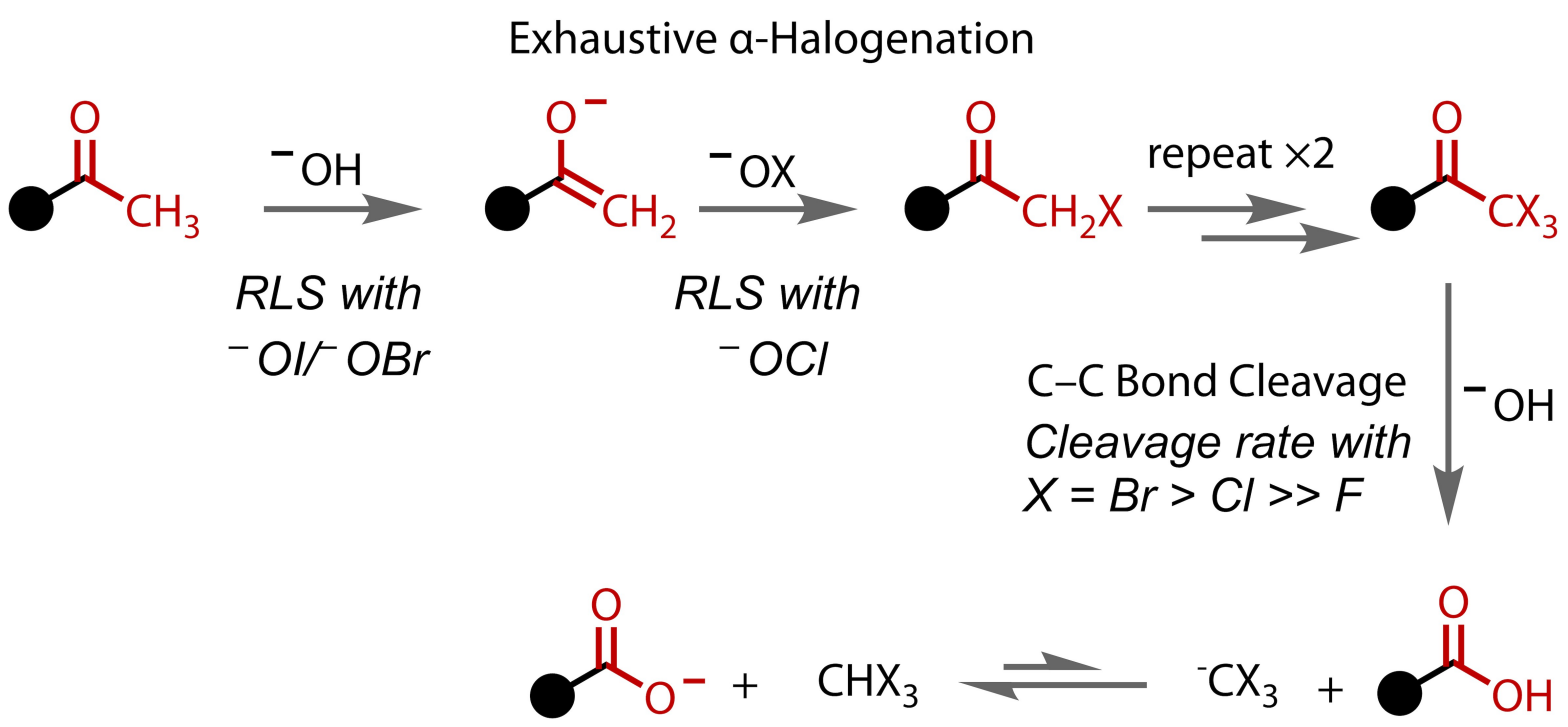
Mechanism of the haloform reaction. RLS=Rate limiting step.

The mechanistic simplicity of the reaction makes it a popular example of carbonyl reactivity in undergraduate chemistry courses and it features in many general organic chemistry textbooks.^[67]^ The mechanism involves two distinct phases: 1) exhaustive α‐halogenation and 2) nucleophilic substitution, resulting in C−C bond cleavage; Figure 5.[^1^, ^17^, ^68^] Base‐catalysed enolisation of a methyl ketone to an enolate, followed by halogenation by a hypohalite yields a halomethyl ketone. Since the monohalogenated ketone is more acidic than the methyl ketone, these steps then repeat until a maximally‐halogenated trihalomethyl ketone is formed. Hydroxyl attack on this reactive species leads to cleavage of the C−CX_3_ bond. Due to the electron‐withdrawing nature of the halogen atoms, the trihalomethyl anion is well‐stabilised. The carboxylic acid product is deprotonated by the trihalomethyl anion, yielding a carboxylate and the haloform species. Isolation of the carboxylic acid is, of course, possible after acidic work‐up.

Based on experiments carried out with the simplest methyl ketone, acetone, the initial enolisation to form the enolate is believed to be the rate‐limiting step with hypobromite or hypoiodite.[^69^, ^70^] With hypochlorite, however, the reaction is orders of magnitude slower, due to rate‐limiting chlorination of enolate to the chloromethyl ketone.^[69]^ The kinetics of the cleavage step of the reaction have also been investigated, with the relative cleavage rates of trihaloacetophenones (PhCOCX_3_) found to follow the order: X=F (1.0)<Cl (5.3×10^10^)< Br (2.2×10^13^).^[71]^ Measurement of the cleavage rate of the analogous triiodoacetophenone was not possible, since triiodomethyl ketone species have never been successfully isolated, although it may be similar to that of the tribromoacetophenone, based on the reported similarity in the acidities of bromoform and iodoform.[^72^, ^73^]

**Figure 6:**
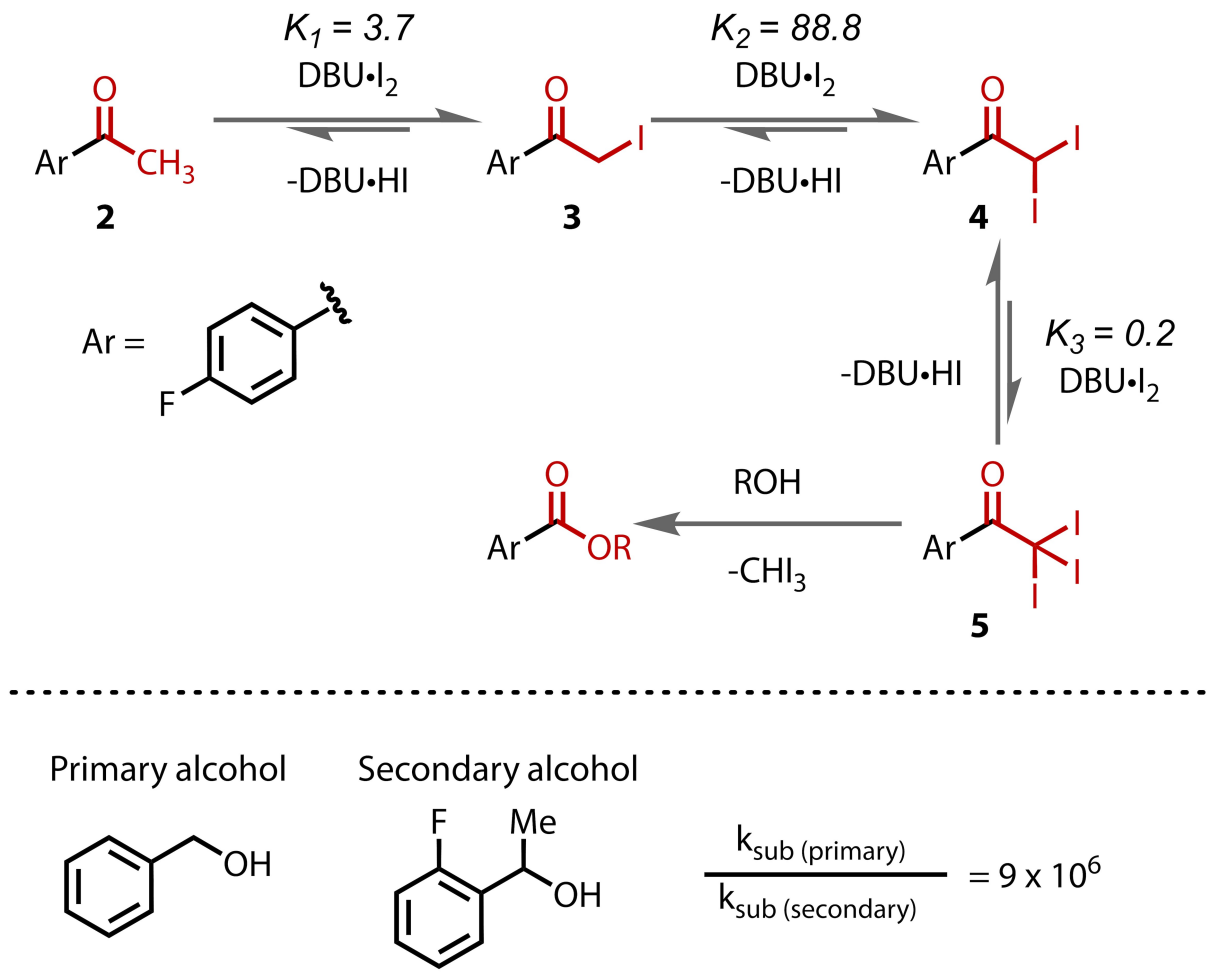
Modelled equilibrium constants for the individual steps in the haloform reaction of 4‐fluoroacetophenone (Ar=4‐F‐C_6_H_4_) with primary and secondary alcohols.

Lennox and co‐workers recently undertook an in‐depth kinetics study of the esterification of methyl ketones in the presence of primary and secondary alcohols.^[74]^ They studied the kinetics of methyl ketone **2** under several sets of conditions and observed that rates of reaction were much slower with secondary alcohols than with primary alcohols, due to the enhanced steric encumbrance in the substitution step. The iodinated intermediates **3**–**5** accumulated and could be observed in the reaction with secondary alcohols, unlike with primary alcohols. A kinetic model supported the discovery that the three iodination steps take place under equilibrium, Figure 6. This previously unreported insight into the haloform reaction mechanism ultimately led to the development of conditions that overcame the significantly weaker nucleophilicity of secondary alcohols relative to primary alcohols to enable ester formation with secondary alcohols.

## 2 Methodology Developments

Historically, hydroxides have been used as the base and elemental halogens as the halonium (“X^+^”) source in the reaction, leading to *in situ* hypohalite formation. Since the earliest reports, the use of safer halide or halite salts in place of molecular halogens have been implemented,^[17]^ as well as the expansion of substrate scopes, the design of reaction conditions that lead to different products and also the use of different starting material moieties. The principles behind these developments are detailed with examples, and sectioned into the type of reaction product that is formed.

### 2.1 Synthesis of Carboxylic Acids

**Figure 7:**
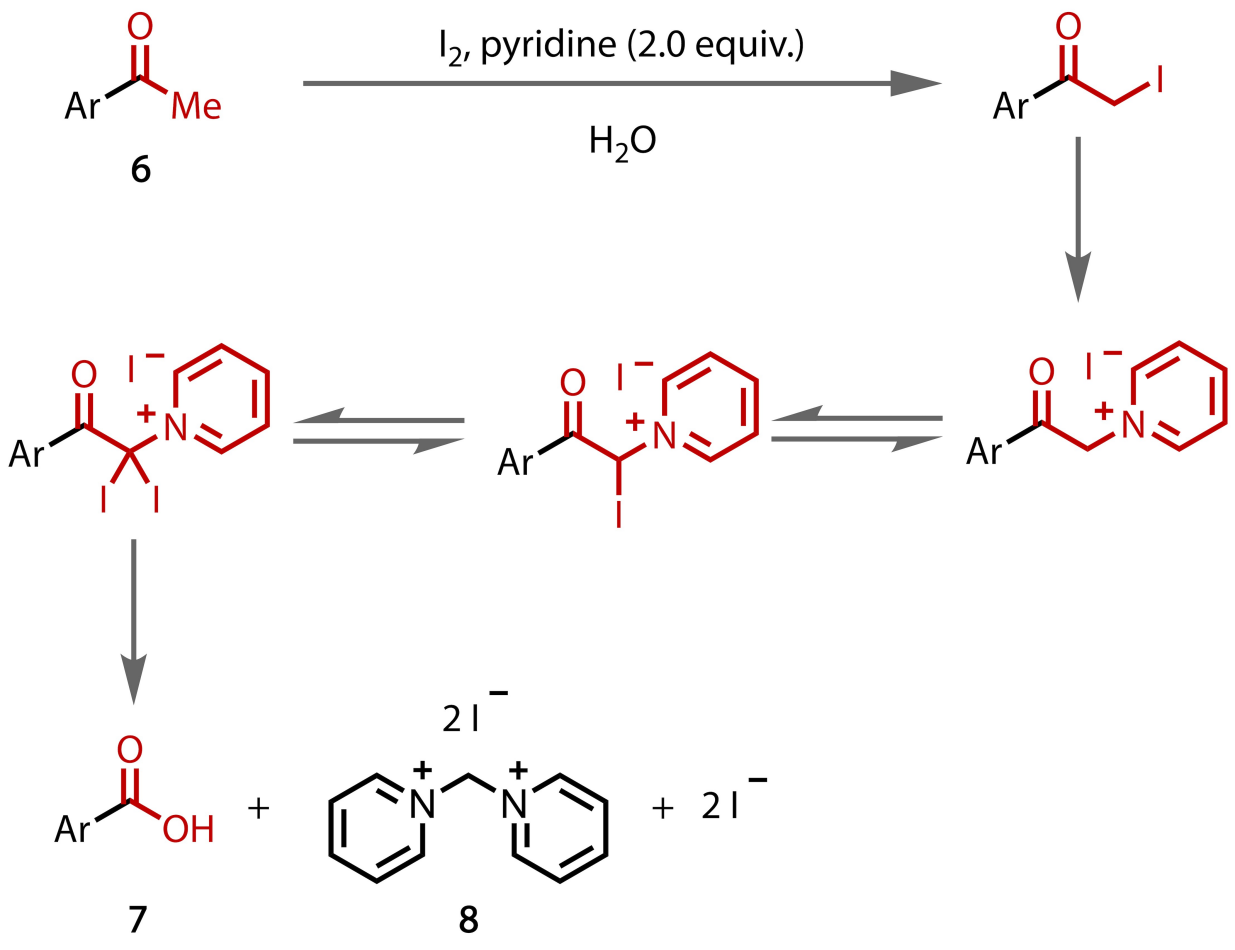
Non classical haloform reaction with iodine and pyridine.

King and Pearson showed in 1946 that the reaction of acetophenone (**6**), iodine and pyridine, or other similar nitrogen bases, could yield benzoic acid (**7**) and *N*,*N*’‐dipyridinium methylene diiodide (**8**), Figure 7.[^75^, ^76^, ^77^, ^78^] The by‐product **8** presumably results from pyridinium‐containing intermediates, which renders the reaction mechanistically very similar to the classical haloform reaction.

**Figure 8:**
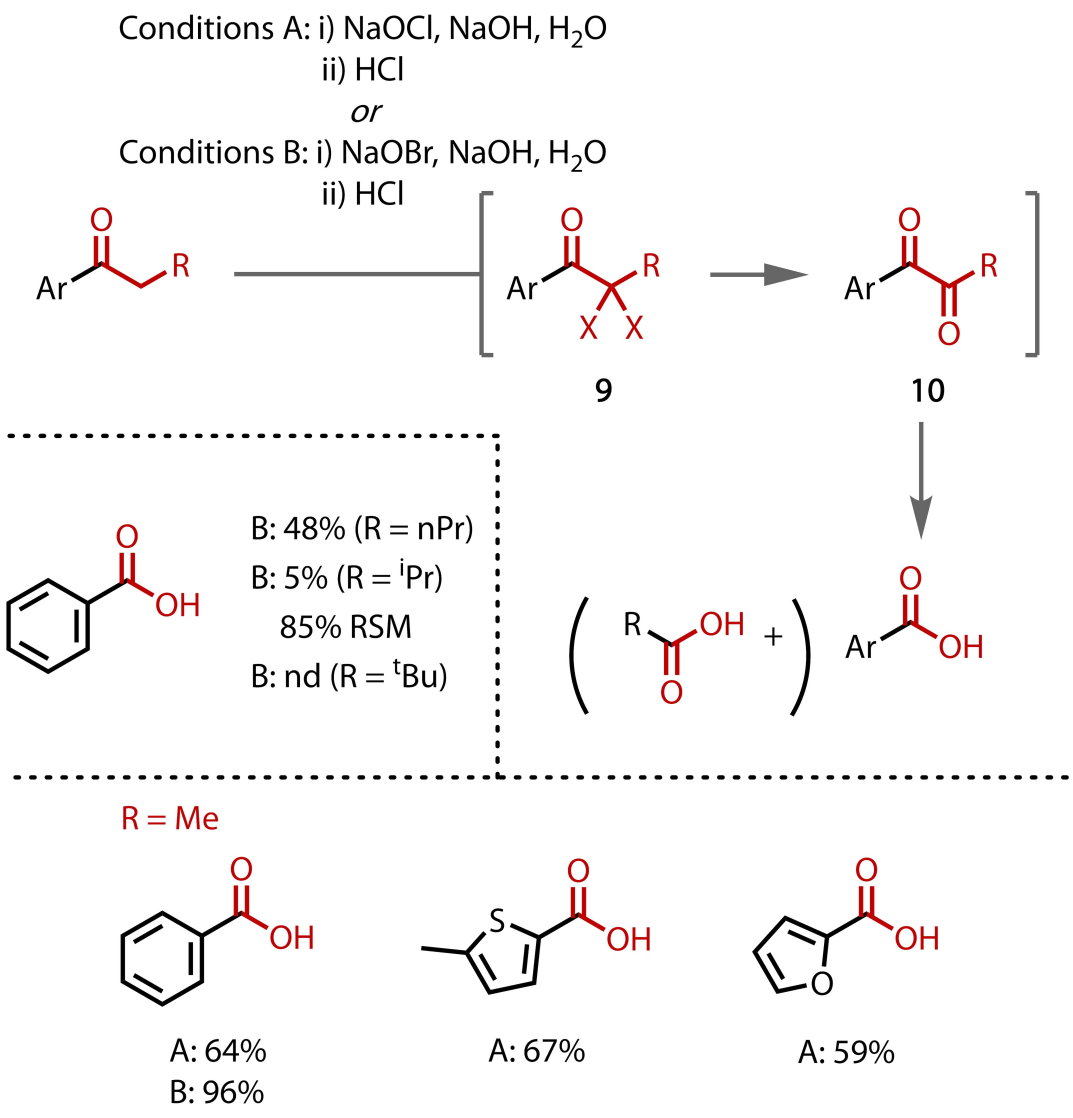
Haloform reaction of higher alkyl (i. e. non methyl) ketones (nd=not detected).

The first definitive synthesis and isolation of carboxylic acids from a haloform reaction with higher alkyl, i. e., non‐methyl, ketones was reported by Farrar and Levine in 1949,^[79]^ although earlier reports of similar reactions appear to have overlooked this significance.[^80^, ^81^] Several aryl/alkyl and heteroaryl/alkyl ketones were converted to their corresponding carboxylic acids, e. g. propiophenone to benzoic acid, in good yields by treatment with alkaline hypochlorite^[79]^ or hypobromite,^[82]^ followed by an acidic workup, Figure 8. The reaction mechanism proposed involves halogenation to an α,α‐dihalo species, which converts to a 1,2‐diketone, followed by cleavage to two carboxylic acids.^[82]^ Since cleavage consumes another equivalent of hypohalite and cannot be achieved with hydroxide alone, formation of an acyl halide intermediate may be involved. This mechanistic proposal is supported by employing **9** or **10** under the reaction conditions, which afforded benzoic acid in 92 % and 91 % respectively. The authors also tested secondary and tertiary alkyl groups attached to the carbonyl group, *iso*‐butyrophenone and pivalophenone respectively, but these were not converted to the corresponding carboxylic acids, suggesting that two hydrogen atoms are required in the alpha position.

**Figure 9:**
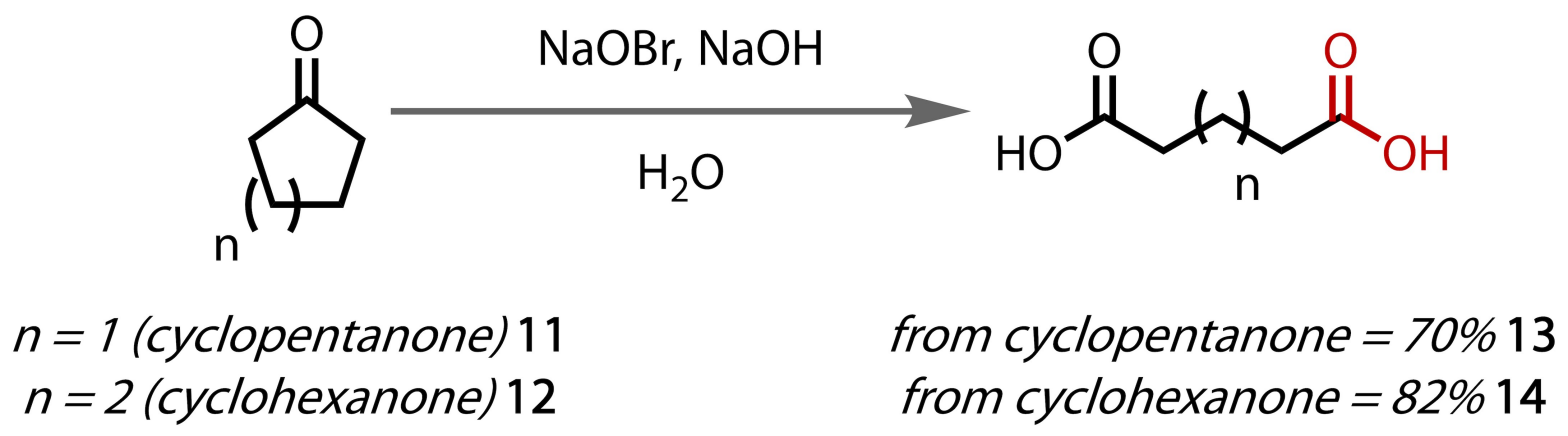
Haloform reaction of cycloalkanones.

An extension of this method to cycloalkanones followed when Farrar reported the synthesis of diacids from cyclopentanone (**11**) and cyclohexanone (**12**) with alkaline sodium hypobromite, Figure 9.^[83]^ During the oxidative cleavage of cyclopentanone, the authors observed significant formation of succinic acid (**13**) instead of glutaric acid (**14**). The selective formation of glutaric acid was optimised by using low temperature and slow addition of the ketone. Cyclic aliphatic ketones display a higher reactivity than their corresponding open‐chain compounds.

**Figure 10:**
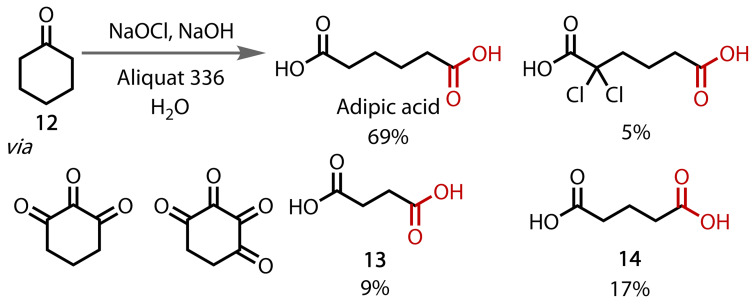
The use of a phase‐transfer catalyst enables a double haloform reaction to produce diacids.

The analogous reaction with hypochlorite has also been reported, in which tetraalkylammonium halide species were employed as phase‐transfer catalysts, Figure 10.^[84]^ The authors exemplified the reaction with cyclopentanone, cyclohexanone, and cyclooctanone using trioctylmethyl ammonium chloride (Aliquat 336) as the phase‐transfer catalyst. An extensive study on cyclohexanone (**12**) was found to afford multiple diacids upon oxidative cleavage, dependent on the pH of the solution. The authors proposed the sequential formation of polyketones to afford succinic (**13**) and glutaric acid (**14**).

**Figure 11:**
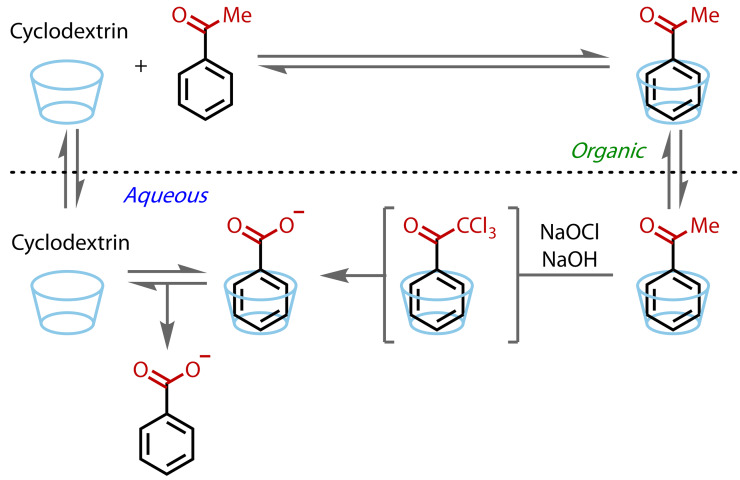
Haloform reaction under inverse phase transfer catalysis conditions with cyclodextrins.

The classical haloform reaction involves aqueous solvent, but methyl ketones are often poorly soluble in water and the halogenated intermediates produced during the reaction are even more hydrophobic. In 2000, Trotta and co‐workers reported on their attempts to address this issue using cyclodextrins as inverse phase‐transfer catalysts.^[85]^ The apolar cyclodextrin cavity helps dissolve the lipophilic reaction species in the aqueous phase, where the haloform reaction occurs and which contains the hypohalite, Figure 11. This enabled modest increases (up to threefold) in the reaction rate of acetophenone with sodium hypochlorite, although a more pronounced catalytic effect was observed with 2‐acetonaphthone, due to its lower water solubility.

**Figure 12:**
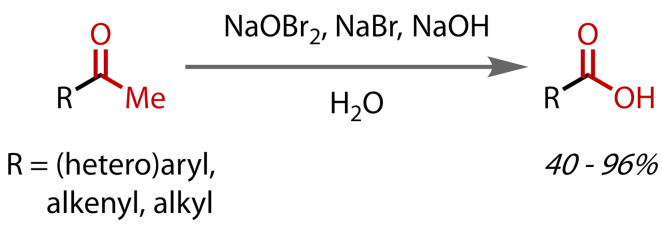
Haloform reaction with sodium bromite.

Alternatives to the classical hypohalite preparations have also been reported. In 1985, Kajigaeshi and co‐workers demonstrated that a mixture of sodium bromite (NaOBr_2_) and sodium bromide in aqueous sodium hydroxide could affect haloform reactions with a range of aryl, alkyl and alkenyl methyl ketones, generally achieving good yields, Figure 12.^[86]^ Although *in situ‐*formed hypobromite (BrO^−^) was believed to be the active species, as a stable, crystalline solid, sodium bromite was proposed to be a practical alternative to molecular halogens for preparing such solutions. With elevated temperatures and extended reaction times, secondary alcohols could also be oxidised to methyl ketones, and then further to carboxylic acids.

**Figure 13:**
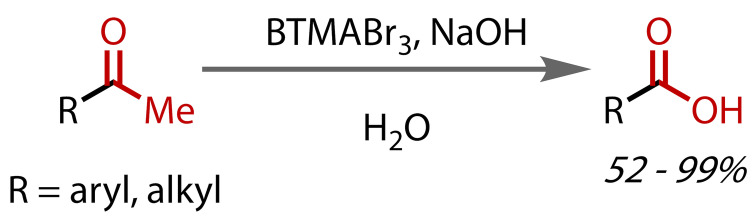
Haloform reaction with BTMABr_3_ as a substitute for bromine.

Kajigaeshi and co‐workers reported similar reactivity with benzyltrimethylammonium tribromide (BTMABr_3_) as a drop‐in replacement for bromine in bromoform reactions in aqueous sodium hydroxide, Figure 13.[^87^, ^88^] BTMABr_3_ is a relatively non‐hazardous, commercially‐available solid, and is therefore an attractive alternative to using elemental bromine. A range of (hetero)aromatic carboxylic acids were afforded in good yields. Under similar conditions, the synthesis of carboxylic acids from primary benzyl alcohols was also possible.^[89]^

**Figure 14:**
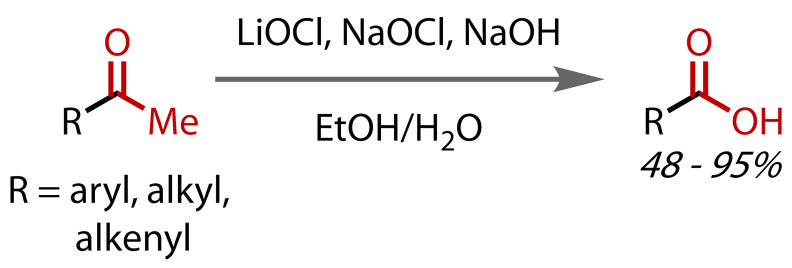
Haloform reaction with a combination of lithium hypochlorite and sodium hypochlorite.

Berlin and co‐workers demonstrated that a combination of lithium hypochlorite, which is readily available as a pool oxidant/cleaner, and sodium hypochlorite bleach could be used to effect haloform reactions, Figure 14.^[90]^ The lithium cation was proposed to coordinate better than a sodium cation to the carbonyl group, leading to more acidic α‐protons, and thus increased reactivity compared to the use of sodium hypochlorite alone. Carboxylic acid synthesis was reported with a range of aryl, alkyl and alkenyl methyl ketones, as well as secondary alcohols, via initial oxidation to methyl ketones, and propiophenone.

**Figure 15:**
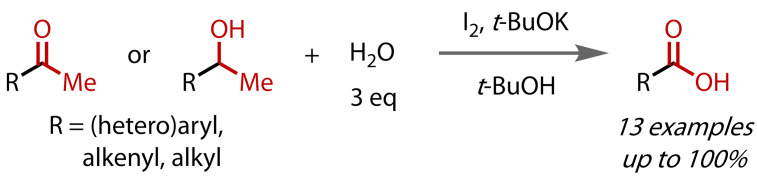
Haloform reaction under non‐aqueous conditions using a reagent quantity of water.

Although the classical haloform reaction calls for aqueous conditions, Uchiyama and co‐workers reported in 2017 the preparation of carboxylic acids with just 3 equivalents of water, Figure 15.^[91]^ Limiting the amount of water used was not the primary focus of the work, rather, the authors were concerned with the synthesis of succinic acid and found that classical haloform conditions suffered from side reactions, resulting in poor yields. Employing an alternative system of iodine and potassium *tert*‐butoxide in *tert*‐butanol, they were able to synthesise a range of aliphatic and aromatic acids in good yields from their corresponding methyl ketones or secondary alcohols (via oxidation to methyl ketones).

**Figure 16:**
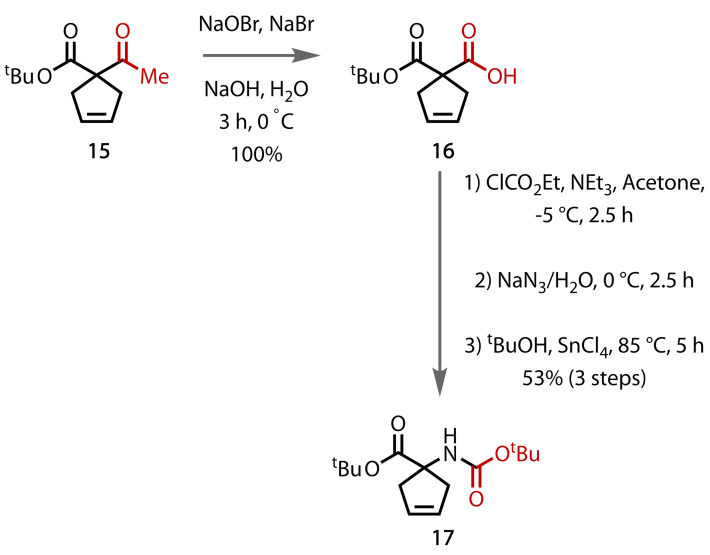
Formation of a carboxylic acid in the presence of an ester.

The chemoselectivity of the haloform reaction was exploited by de Meijere and co‐workers to selectively form a carboxylic acid in the presence of a labile ester, Figure 16.^[92]^ Treatment of β‐ketoester **15** with NaOBr/NaBr in alkaline solution generated a carboxylic acid **16** in quantitative yield, which then underwent a Curtius rearrangement to form amide **17**. In general, the haloform conditions display good functional group tolerance, with no side reactivity on electron‐rich alkenes, aromatic rings or esters.[^17^, ^74^]

### 2.2 Synthesis of Esters

Esters, which are prevalent in pharmaceuticals, natural products[^93^, ^94^] and are used extensively in the flavouring and fragrance industries,^[95]^ can be made via many different methods,^[96]^ most commonly from carboxylic acids, e. g. Fischer^[97]^ and Steglich^[98]^ esterifications. However, their preparation from ketones is much rarer.

While the synthesis of carboxylic acids from ketones via the haloform reaction had been used in a preparative fashion since at least the early 1900s,^[1]^ and the decomposition of trichloromethyl ketones to esters with sodium alkoxides had been known since 1931,^[28]^ the discovery that esters could be directly synthesised by a haloform reaction with methyl ketones, if alcohol was employed as a co‐solvent, was not made until 1944, Figure 17.^[99]^ The isolation of ester **18** was reported upon the action of hypochlorite on methyl ketone **19**. The authors proposed that ester products had not previously been reported due to their facile hydrolysis under the basic reaction conditions, a process that was only avoided in this case by the spontaneous precipitation of the ester.

**Figure 17:**
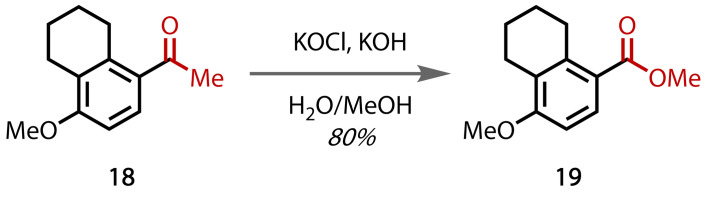
Discovery that esters can be synthesised via the haloform reaction with alcohol cosolvents..

Hypohalite‐based haloform reactions are generally limited to electron‐deficient aryl ketones, as ring‐halogenation of electron‐rich aryl ketones has been observed.^[100]^ Additionally, hypohalite species can oxidise alcohols to aldehydes and ketones, further limiting the substrate scope.[^20^, ^101^] Therefore, research efforts towards esters have sought to alter the leaving group employed.

**Figure 18:**
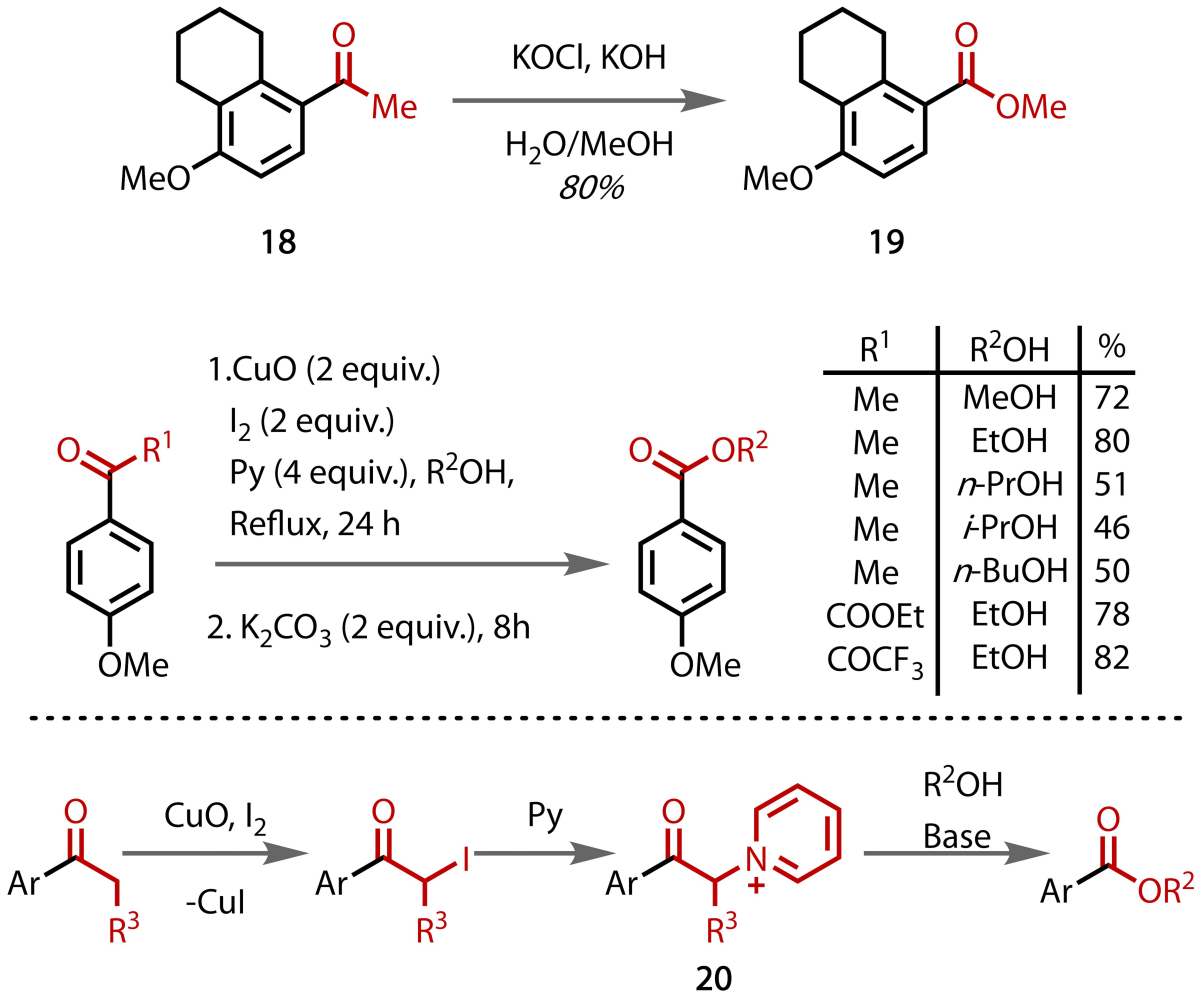
Synthesis of simple alkyl esters via a haloform reaction with iodine and pyridine.

In 2008, Wu and co‐workers reported that esters could be synthesised from acetophenones via a haloform reaction with iodine, pyridine and copper(II) oxide in alcohol solvent, Figure 18.^[102]^ The reaction appears to be mechanistically similar to that reported by King and Pearson,[^77^, ^78^] where initial α‐iodination is followed by substitution to give a pyridinium‐containing intermediate **20**. The scope of the method extended beyond methanol to include ethanol, and, somewhat less successfully, *n*‐propanol, *n*‐butanol and isopropyl alcohol. *tert*‐Butanol was also tested, but the *tert*‐butyl ester was not observed. Esters could also be prepared from β‐ketoesters, 1,3‐diketones and propiophenone, albeit with a reduced yield in the latter instance.

**Figure 19:**
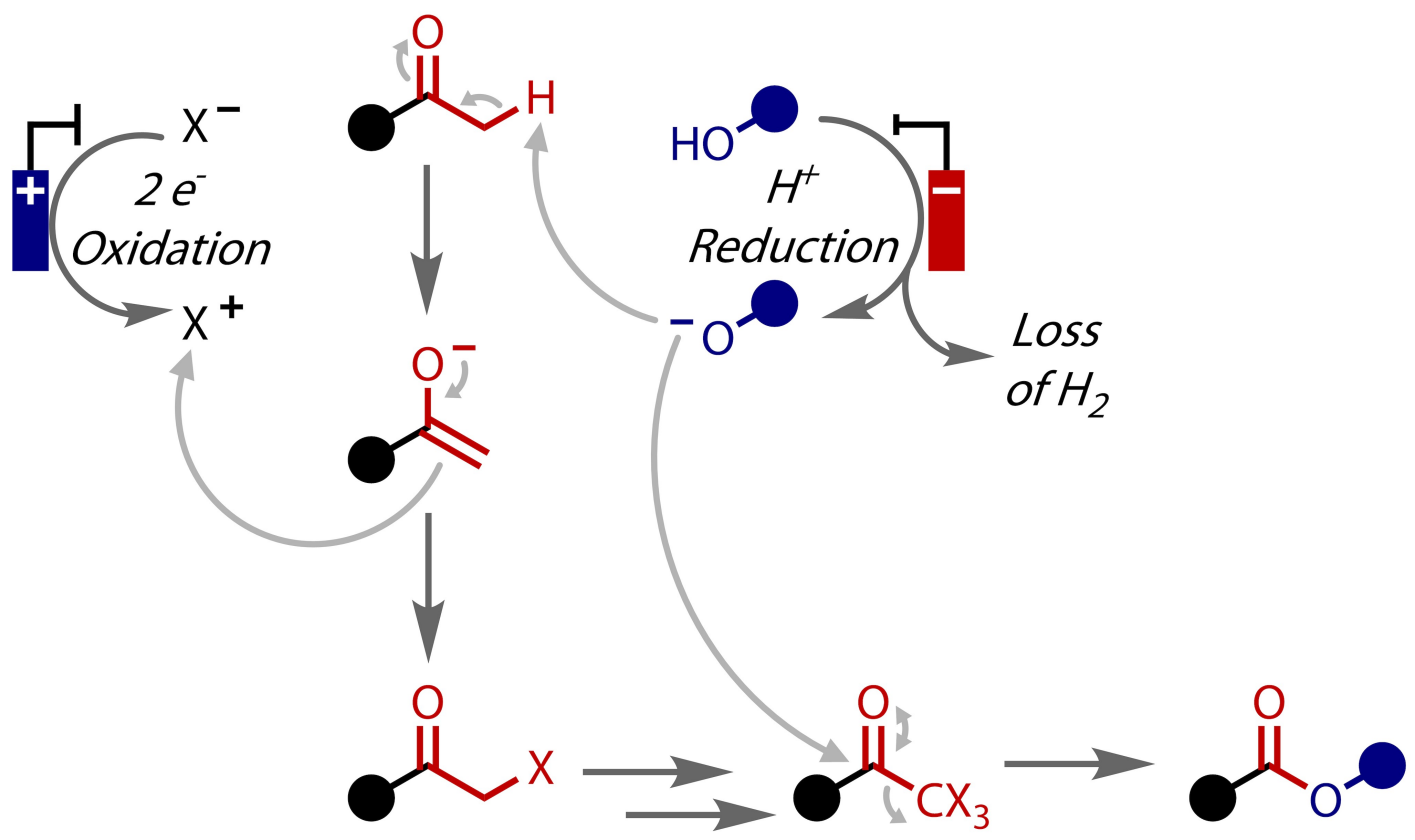
Idealised mechanism for electrochemical haloform synthesis of esters.

Halonium ions can be generated by electrochemical oxidation of inexpensive and comparatively benign halide salts,[^103^, ^104^] which can then be employed to halogenate methyl ketones, producing the key trihalomethyl ketone intermediates, Figure 19. In this scenario, electrodes have replaced the chemical halonium oxidants, demonstrating one of the green chemistry benefits electrochemistry can offer.^[105]^

**Figure 20:**
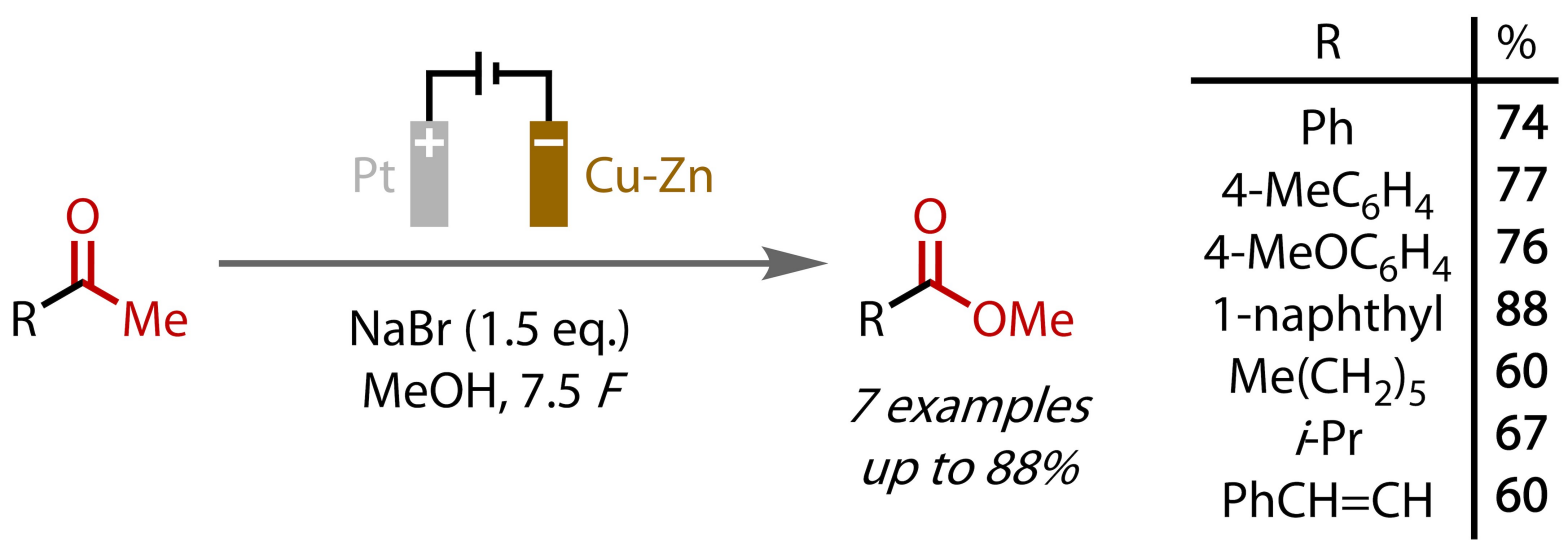
Methyl ester synthesis via electrochemical haloform reaction.

The first such example of an electrochemical haloform reaction was reported by Nikishin and co‐workers in 1988: methyl esters were prepared via bromide oxidation in methanol.[^106^, ^107^, ^108^] Sodium bromide was found to be the best halide source, which oxidised in an undivided cell at a platinum anode with a brass cathode, and enabled the conversion of aryl, alkyl and alkenyl methyl ketones to their corresponding methyl esters in good yields, Figure 20. The reaction was able to proceed with catalytic quantities of bromide (<3 equivalents), as it was regenerated from the bromoform via reaction with methoxide or, to a lesser extent, cathodic reduction. A modest excess of charge (7.5 *F* vs 6 *F* for 100 % Faradaic efficiency) was required to obtain optimal yields.

**Figure 21:**
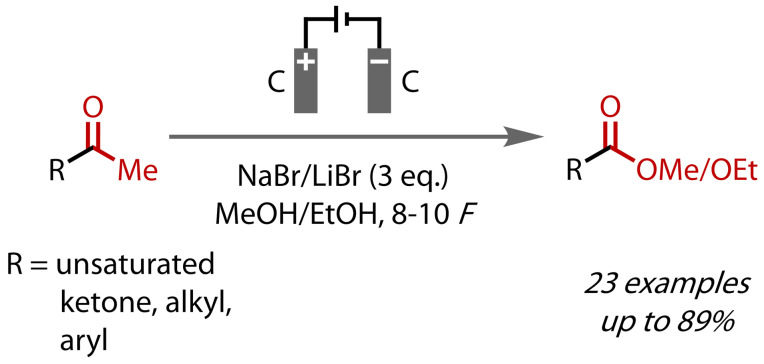
Synthesis of methyl and ethyl esters via electrochemical haloform reaction.

Nishiguchi and co‐workers reported a very similar method of electrochemical ester synthesis in 1996, in this case preparing both methyl and ethyl esters and with an expanded ketone scope, which included cyclic 1,3‐diketones, Figure 21.^[109]^ Consistently high yields were reported for the conversion of a range of alkyl, α,β‐unsaturated and aryl methyl ketones. Sodium bromide was again used as the bromination source in methanol, but lithium bromide was preferred for electrolyses in ethanol, due to its superior solubility. The conditions employed by Nishiguchi were otherwise broadly similar to those used by Nikishin, although carbon rods were employed as electrodes and slightly more charge (8–10 *F*) was passed. Notably, the authors emphasised the need for anhydrous alcohol solvent to prevent the formation of carboxylic acid side products.

**Figure 22:**
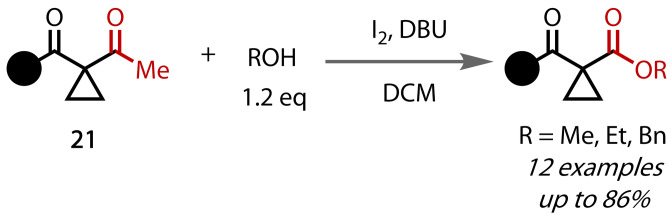
Haloform reaction with stoichiometric alcohols. Bn=Benzyl.

Zhang, Dong and co‐workers described in 2012 the conversion of a small group of acetylcyclopropanes (**21**) to their corresponding cyclopropyl esters with just 1.2 equivalents of alcohol in DCM, Figure 22.^[110]^ Molecular iodine and 1,8‐diazabicyclo[5.4.0]undec‐7‐ene (DBU) replaced the hypohalite of the classical haloform reaction. The scope of the reaction was limited to only three simple primary alcohols, methanol, ethanol and benzyl alcohol, and only cyclopropanoate esters bearing β‐amido or ‐keto groups were synthesised.

**Figure 23:**
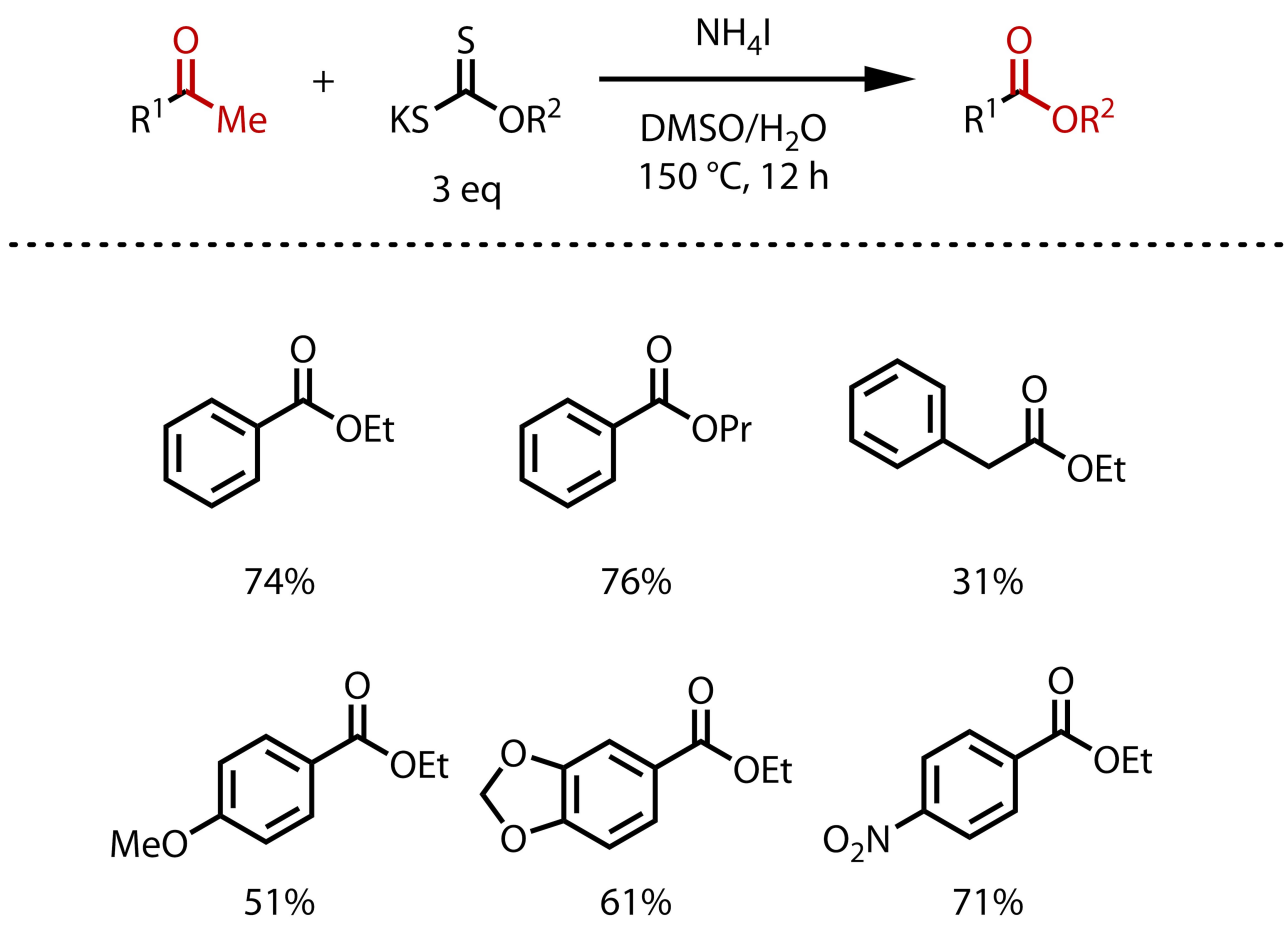
Synthesis of ethyl and propyl esters via haloform reaction with xanthate reagents.

In 2020, Huang and Li reported a method that dispensed with the need for alcohol solvent, instead using potassium xanthates, prepared from alcohols and carbon disulfide, as the alkoxy source, Figure 23.^[111]^ A radical mechanism was proposed, invoking a classical triiodomethyl ketone intermediate *en route* to the ester. This enabled the synthesis of esters from a range of predominantly acetophenones with ammonium iodide in a solvent mixture of DMSO and water. Ethyl and propyl esters were synthesised, although, in principle the method could be extended by preparing xanthates with other alcohols.

**Figure 24:**
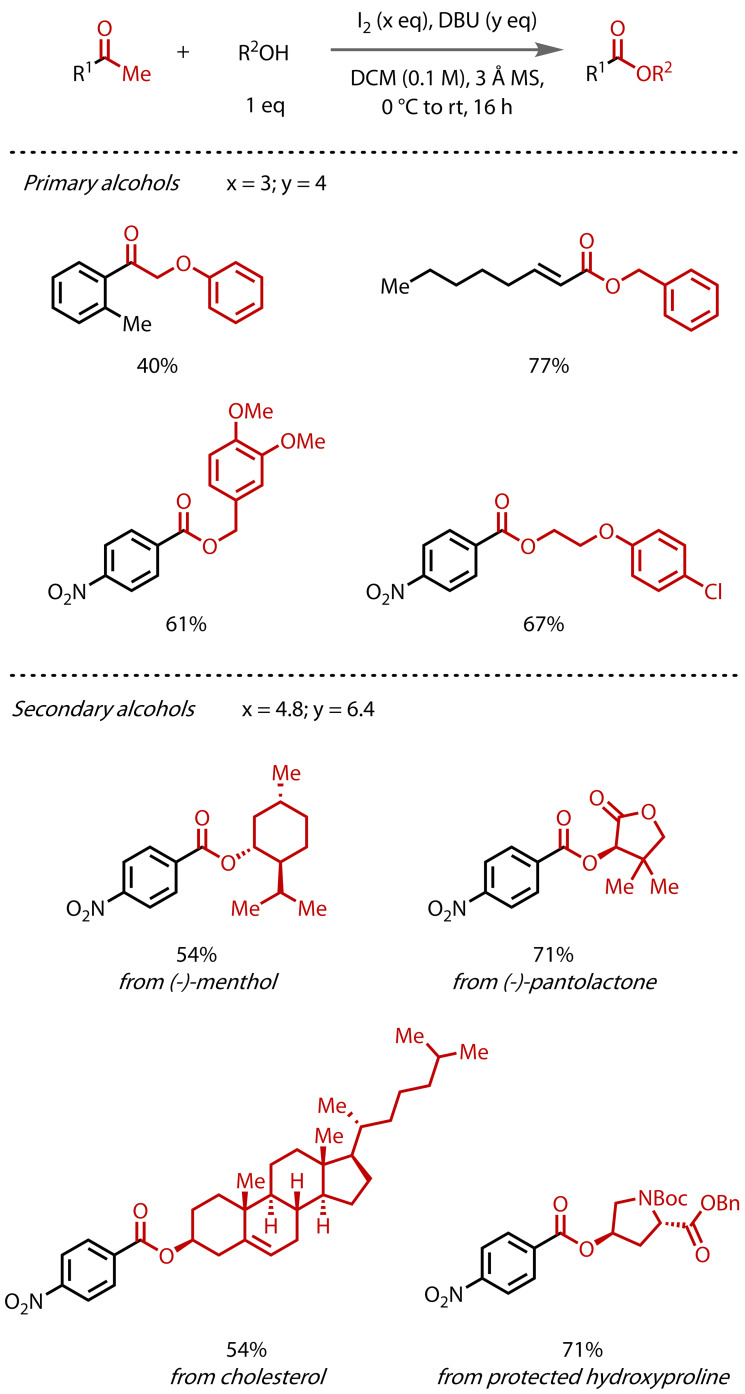
General “haloform coupling” reaction, with selected examples involving both primary and secondary alcohols.

In 2024, Lennox and co‐workers reported a general method for ester synthesis via the haloform reaction with 1.0 equivalents of either primary or secondary alcohols, Figure 24.^[74]^ This study addressed the two main issues with the classical haloform reaction as a method for ester synthesis: 1) the requirement for solvent‐level alcohol, which limits the scope of esters that can be accessed in an economically‐viable manner; and 2) the limitation for primary alcohols only. To avoid competing carboxylic acid formation, they employed anhydrous conditions and reagents, including DBU and iodine. This ‘haloform coupling’ reaction was found to be compatible with a wide range of complex primary alcohols. However, the use of secondary alcohols was not immediately viable under the conditions for primary alcohols, with only low yields obtained. However, in‐depth kinetic modelling studies revealed that iodination steps were in equilibrium, which led to a change in the conditions for secondary alcohols. By increasing the concentrations of both iodine and DBU, the equilibrium could be pushed to the triiodo intermediate, increasing its concentration and thereby the reaction rate of substitution. This subtle change led to a significant increase in reaction yields with secondary alcohols, enabling the direct construction of valuable, structurally‐complex esters that were previously inaccessible via the haloform reaction.

### 2.3 Synthesis of Amides

**Figure 25:**
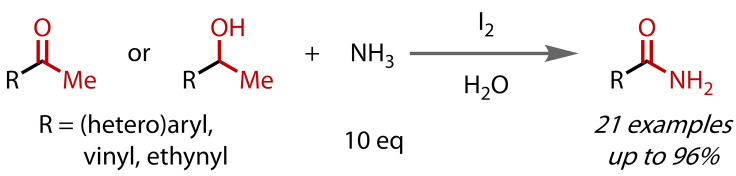
Primary amide synthesis via haloform reaction with aqueous ammonia.

A methodology to prepare amides via a classic haloform‐type reaction mechanism was described in 2009 by Wu and co‐workers, who reported the synthesis of aryl, heteroaryl and alkenyl (α,β‐unsaturated) primary amides by employing aqueous ammonia as the nitrogen source, Figure 25.^[112]^ Since 10 equivalents of ammonia were used, no additional base was required. The method was successfully applied to several 1‐arylethanols. Good yields were obtained with (hetero)aryl methyl ketones and 1‐arylethanol substrates, demonstrating that, like in the classical haloform reaction, alcohol to ketone oxidation prior to the halogenation and cleavage sequence, was possible under the reaction conditions. Vinyl and ethynyl ketones were also transformed, albeit in slightly lower yields. In contrast to the ester‐forming reactions, this reaction and several other amide‐forming reactions take place in water. This contributes to the success since the iodoform by‐product is insoluble in the aqueous medium and its precipitation drives the reaction.

**Figure 26:**
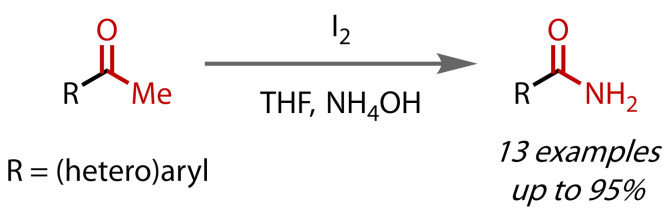
Primary amide synthesis using aqueous ammonia and THF.

After this initial report, several similar methods rapidly followed. Cuevas‐Yañez and co‐workers reported the synthesis of a range of primary aryl‐ and heteroarylamides from their respective ketones with iodine in aqueous ammonia and a small amount of THF cosolvent, Figure 26.^[113]^ Yields of arylamides were very good but slightly lower for heteroaryl amides. Notably, nitrogen heterocycle‐containing substrates were tolerated, which were unsuccessful under Wu's conditions.^[112]^ Two plausible mechanisms were proposed: triiodination followed by substitution with NH_3_ or enamine formation, triiodination, then tautomerisation.

Togo and co‐workers also used iodine in aqueous ammonia, this time in acetonitrile and following a Friedel‐Crafts acylation of the arenes. The regioselectivity of the acylation was imperfect with some arenes and additional ring bromination was observed with some ethyl(hetero)arenes. Nevertheless, this two‐step, one‐pot methodology enabled access to primary aryl‐ and heteroaryl‐ amides from (hetero)arenes^[114]^ and ethyl(hetero)arenes^[115]^ in generally good yields.

**Figure 27:**
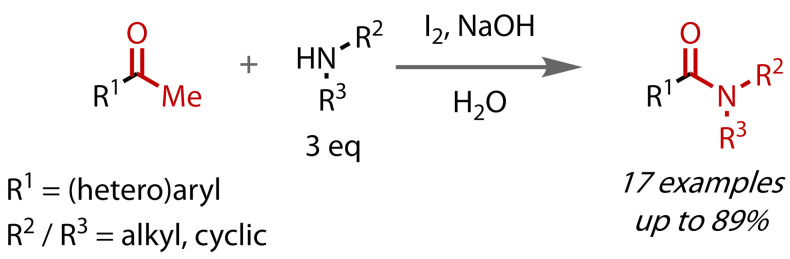
Secondary and tertiary amide synthesis via haloform reaction with stoichiometric amines.

In 2011, Wu and co‐workers extended their original amidation conditions to access secondary and tertiary amides, Figure 27.^[116]^ Using iodine and sodium hydroxide in water, to generate hypoiodite *in situ*, aryl and heteroaryl methyl ketones were coupled with primary and secondary amines, demonstrating that secondary and tertiary amide synthesis is possible under classical haloform conditions. Since only three equivalents of, albeit relatively simple, aliphatic primary and secondary amines were coupled, this is the most significant example of a stoichiometric amide haloform reaction reported to date. The yields obtained were higher with primary amines than with the more sterically‐hindered secondary amines, but were generally lower than the yields of the corresponding primary amides previously reported.^[112]^ Reported limitations included aniline, which was insufficiently nucleophilic, and *t*‐butylamine, which was too bulky. Again, the insolubility of iodoform in water served to promote the transformation.

**Figure 28:**
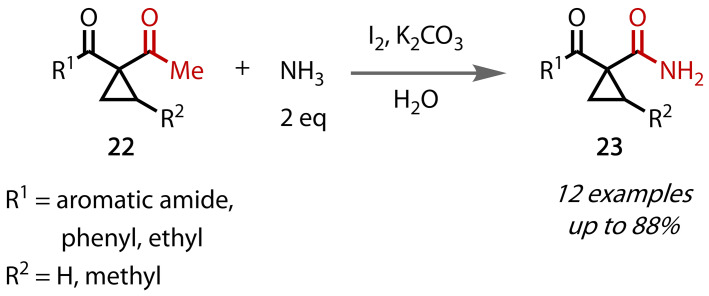
Primary amide synthesis via haloform reaction with stoichiometric aqueous ammonia and additional base.

An alternative method for the preparation of primary amides was reported by Zhang, Dong and co‐workers in 2012.^[110]^ Motivated by a desire to access substituted cyclopropane building blocks, the authors reported conditions very similar to those originally used by Wu, but with the addition of aqueous potassium carbonate, which enabled the use of just two equivalents of aqueous ammonia. The efficacy of these conditions was demonstrated by the conversion of a series of acetylcyclopropanes (**22**) to cyclopropyl amides (**23**), Figure 28. Yields of the cyclopropyl amides were typically good to excellent. The products were demonstrated to be viable substrates for further manipulation, with the amide converted to a cyano group under Vilsmeier conditions.

**Figure 29:**
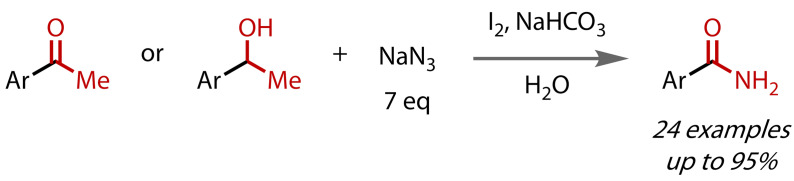
Primary amide synthesis via a haloform reaction with stoichiometric azide as used as the source of nitrogen.

Narender and co‐workers reported in 2013 that sodium azide could be used as an alternative nitrogen source in the synthesis of primary amides, Figure 29.^[117]^ Attack by azide on the triiodomethyl ketone intermediate followed by loss of nitrogen gas yielded primary aryl‐ and heteroarylamides in good to excellent yields. Two benzoxazolone drug structures were accessed in a single step. Their reaction system was otherwise unremarkable: a mixture of iodine and sodium bicarbonate in water.

**Figure 30:**
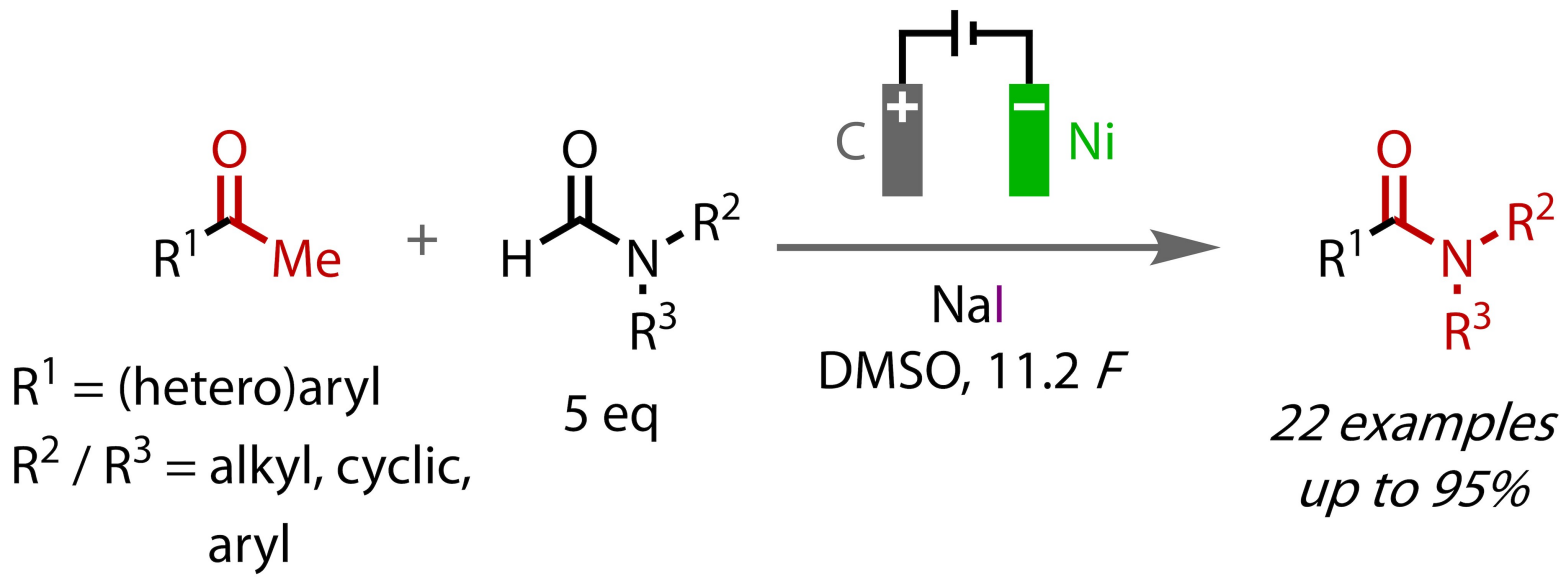
Amide synthesis via electrochemical haloform reaction with formamides.

In 2013, Huang *et al*. used electrochemical oxidation to generate iodine from sodium iodide to form a range of secondary and tertiary (hetero)aryl amides that were synthesised in good to excellent yields, Figure 30.^[118]^ During this paired electrolysis, iodine is generated at a graphite anode, while formamide is reduced to yield the amine at a nickel cathode. Amide formation ensues when the amine attacks the triiodomethyl ketone intermediate generated, since both processes occur in the same solution in an undivided cell. Interestingly, paired electrolysis using 5 equivalents of formamide gave a better yield than simply using the corresponding amine, presumably due to deleterious cathodic reactions that take place in the latter case. Sodium iodide was found to be the optimal iodine source, which also serves as a supporting electrolyte, while a small amount of water was found to be crucial for the reaction, with only trace product detected using dry DMSO. Limitations of the method include its relatively low Faradaic efficiency due to reduction of DMSO to dimethyl sulfone as a side reaction, and the lower yields achieved using bulkier formamides.

**Figure 31:**
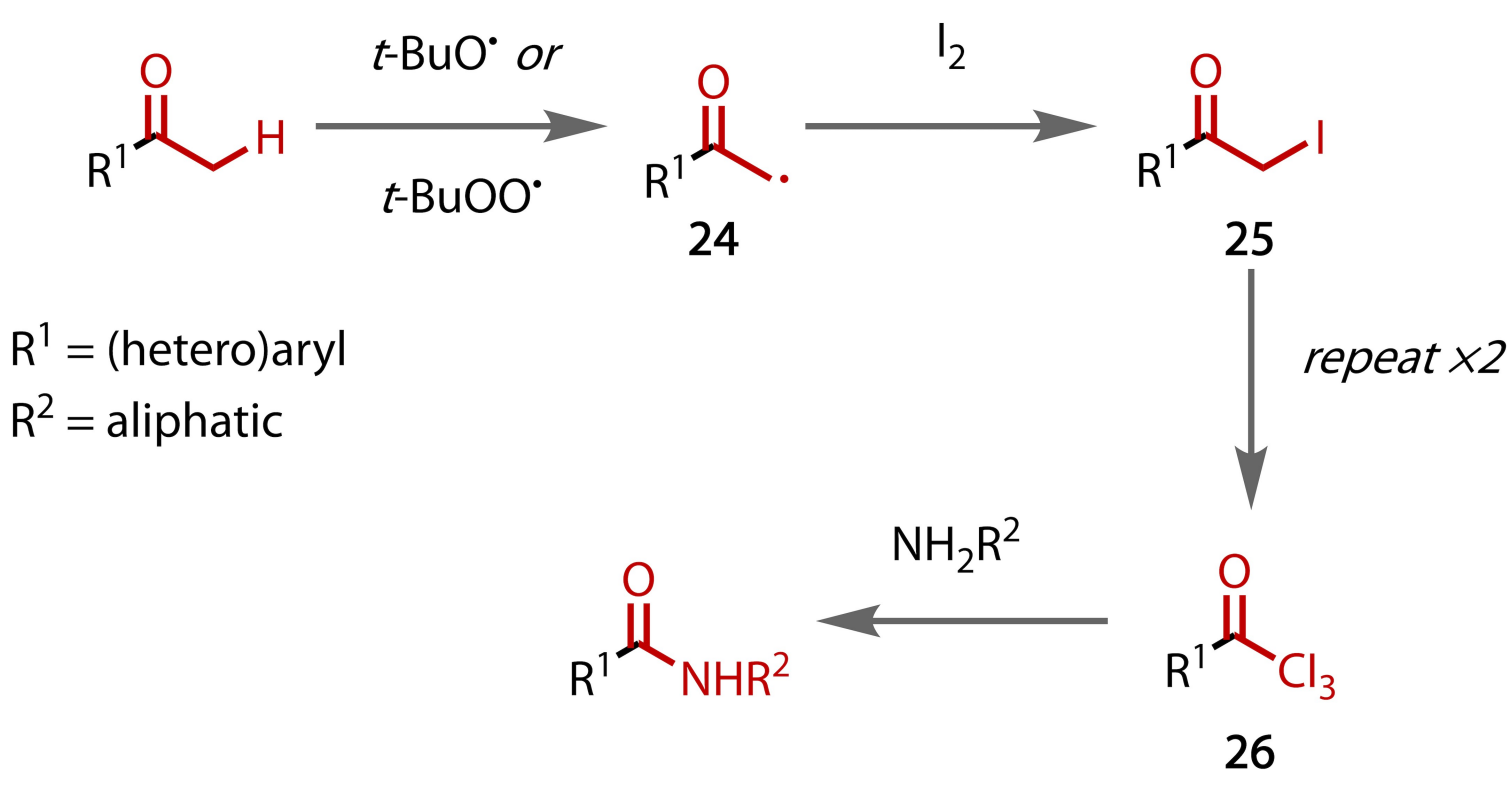
Proposed radical pathway for haloform reaction with sub‐stoichiometric iodine and TBHP.

In 2016, Bathula and co‐workers reported a method that employed catalytic iodine (30 mol % vs ketone) that was proposed to regenerate via oxidation of iodide with DMSO.^[119]^ However, they do not propose a typical haloform mechanism. Similar approaches to forming primary and secondary amides using sub‐stoichiometric iodine have been reported by Chaskar,^[120]^ and Wan and Zhu,^[121]^ respectively, who regenerated the iodine with *tert*‐butyl hydroperoxide (TBHP). While Chaskar's method employed ethylarenes,^[120]^ both sets of conditions were proposed to proceed via triiodomethyl ketone intermediates, with Wan and Zhu having confirmed the presence of iodoform as a by‐product in their reaction mixtures.^[121]^ Although only circumstantial evidence was provided, Wan and Zhu suggested that iodination may proceed via a radical pathway, with TBHP generating a methylene radical (**24**), which could then abstract an iodine atom to form an iodomethyl ketone intermediate (**25**), Figure 31. Repetition of this process would generate the triiodomethyl ketone (**26**), which could be cleaved in the normal manner to yield the amide.

Wu and Beller reported similar conditions that combined catalytic TBAI with TBHP to transform acetophenones to benzamides,^[122]^ although an alternative mechanism, not based on the haloform reaction, was proposed. The method was applied to the synthesis of a wide range of (hetero)aryl amides from their corresponding (hetero)aryl methyl ketones and carbinols in moderate to good yields, as well as an α,β‐unsaturated methyl ketone and two propiophenone substrates.

**Figure 32:**
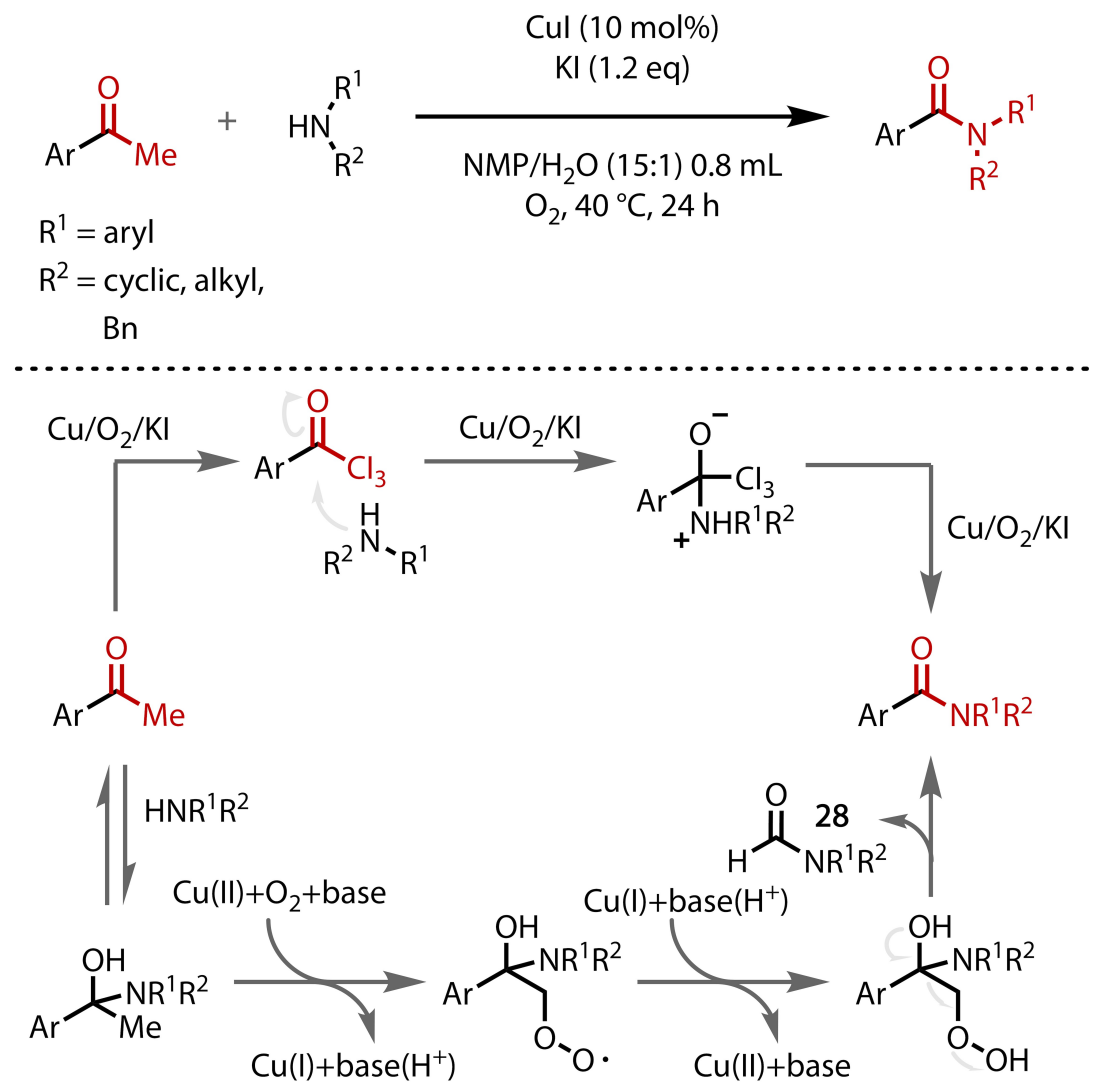
Two proposed mechanisms for the reaction with CuI and KI. Bn=Benzyl.

Lei and co‐workers reported a haloform‐like synthesis of tertiary amides, using catalytic CuI and KI in a mixture of NMP and water.^[123]^ Moderate to good yields were recorded for the conversion of a range of acetophenones to tertiary amides with four different secondary amines, Figure 32. Whether these conditions lead to a reaction that can be classified as a haloform reaction or simply as having ‘haloform‐like’ reactivity is uncertain. A classic haloform mechanism is presented as being a possibility, with the triiodination achieved in the presence of Cu/O_2_/KI. However, an alternative mechanism is also proposed that involves the incorporation of O_2_ into a reaction intermediate (**27**), which also explains the observed formation of a formamide side product (**28**).

**Figure 33:**
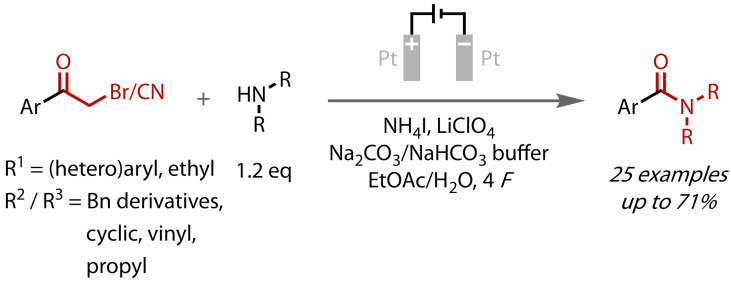
Amide synthesis via electrochemical haloform reaction with amines. Bn=Benzyl.

Gu and Li reported the use of α‐bromo‐ or ‐cyanomethyl ketones as suitable starting materials for an amide‐forming haloform reaction.^[124]^ Through single examples, 1,3‐diketone and β‐keto ester substrates were also shown to be viable substrates, albeit with reduced yields. They employed an electrochemical approach with free amines used in only a very slight excess (1.2 equivalents), Figure 33. Ketone iodination was achieved via iodide oxidation and facilitated by an aqueous carbonate buffer. This buffer created a biphasic system with the ethyl acetate cosolvent and resulted in significant carboxylic acid formation as a side reaction (19 % with the model substrate). That amides were still the major products, however, combined with Yuan and co‐workers’ observation that *“a small amount of water was crucial for this reaction”*,^[118]^ demonstrates the relatively facile nature of amide coupling compared to ester coupling.

**Figure 34:**
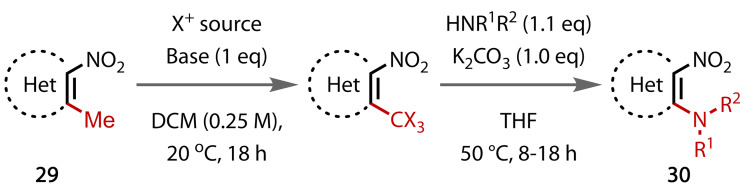
Haloform‐type aromatic amination. Het=Heterocycle.

Recently Monasterolo and Adamo reported an interesting ‘vinylogous nitro‐haloform’ reaction that transformed aminated methyl‐isoxazoles (**29**), Figure 34.^[125]^ The methyl group was first halogenated with electrophilic bromine or chlorine sources. Then substitution with a primary or secondary amine at the electrophilic carbon displaces a haloform by‐product. This two‐step methodology forms amine (**30**) products, as opposed to amides, but is mechanistically aligned to a classical haloform reaction because of the similarity in approach. This report raises the question of whether other methyl groups could be displaced using an approach of exhaustive halogenation.

## 3 Applications in Synthesis

In this section, an overview of where the haloform reaction has been used in the synthesis of complex organic molecules is provided. Particular emphasis is placed on applications in natural product synthesis, where the strategic use of the haloform reaction within a whole synthetic route can be evaluated.

**Figure 35:**
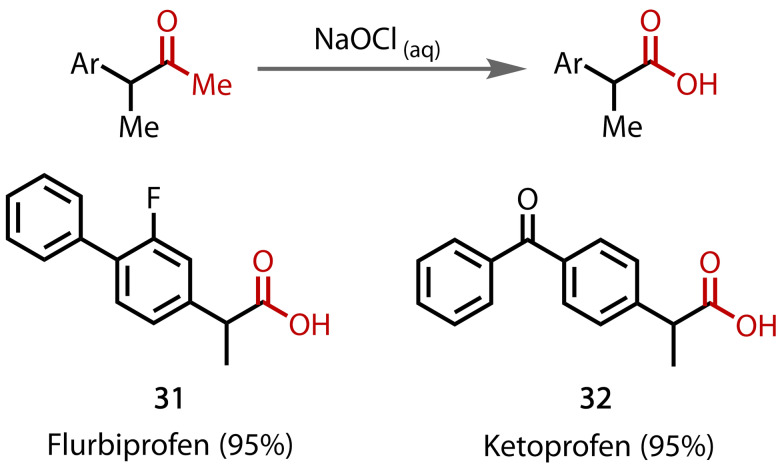
The haloform reaction used in the formation of 2‐arylpropanoic acids (31, 32).

In medicinal chemistry, the haloform reaction has found applications in the synthesis of common drug scaffolds. Frank and co‐workers used the haloform reaction as a key step in the structural modification of steroidal inhibitors based on the natural product, pregnenolone, and utilised the resultant carboxylic acid for further diversifications.^[126]^ The reaction has also been used in lead optimisation,^[127]^ structure‐activity relationship studies,^[128]^ and in the synthesis of pharmaceutically active 2‐arylpropanoic acids (**31**, **32**),^[129]^ e. g., in flurbiprofen and ketoprofen, Figure 35.

**Figure 36:**
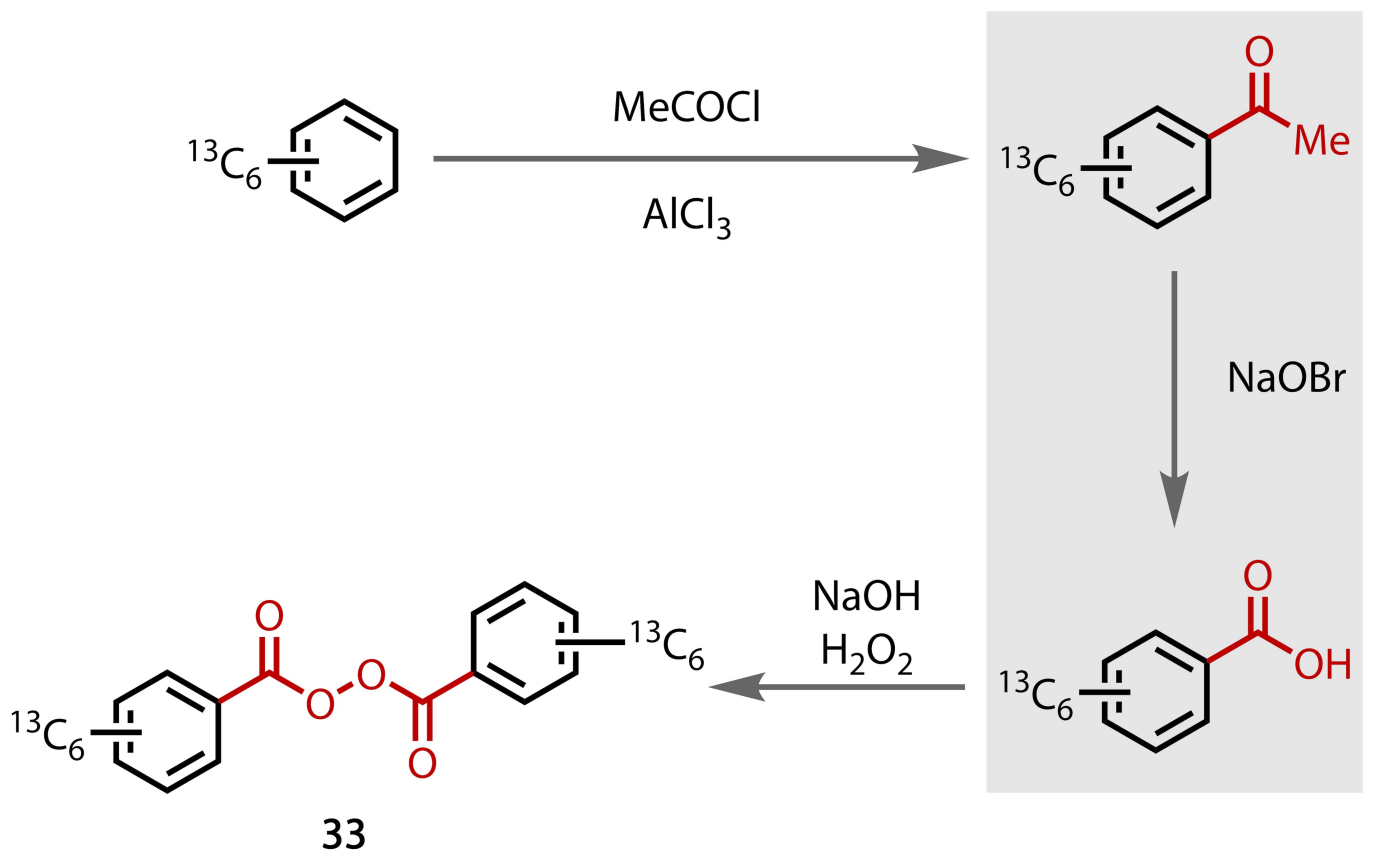
Synthesis of ^13^C_12_‐benzoyl‐peroxide (33) via the haloform reaction.

Isotopically‐labelled compounds, including ^13^C_12_‐benzoyl peroxide **33**, Figure 36, have been prepared using the haloform reaction in combination with a Friedel‐Crafts acylation to install the methyl ketone.^[130]^

The haloform reaction has also been used as a method for derivatising the chiral pool, for example, in structure‐activity relationship studies of chiral ionone alkaloids for the treatment of breast cancer, where the racemic compound was previously found to have a significant inhibitory effect.^[131]^ However, it is now known that a single enantiomer possesses the biological effect, so a route to the single enantiomer is required. The haloform reaction was exploited as a route for the chiral resolution of the carboxylic acid derived from commercially available α‐ionone.^[132]^

**Figure 37:**
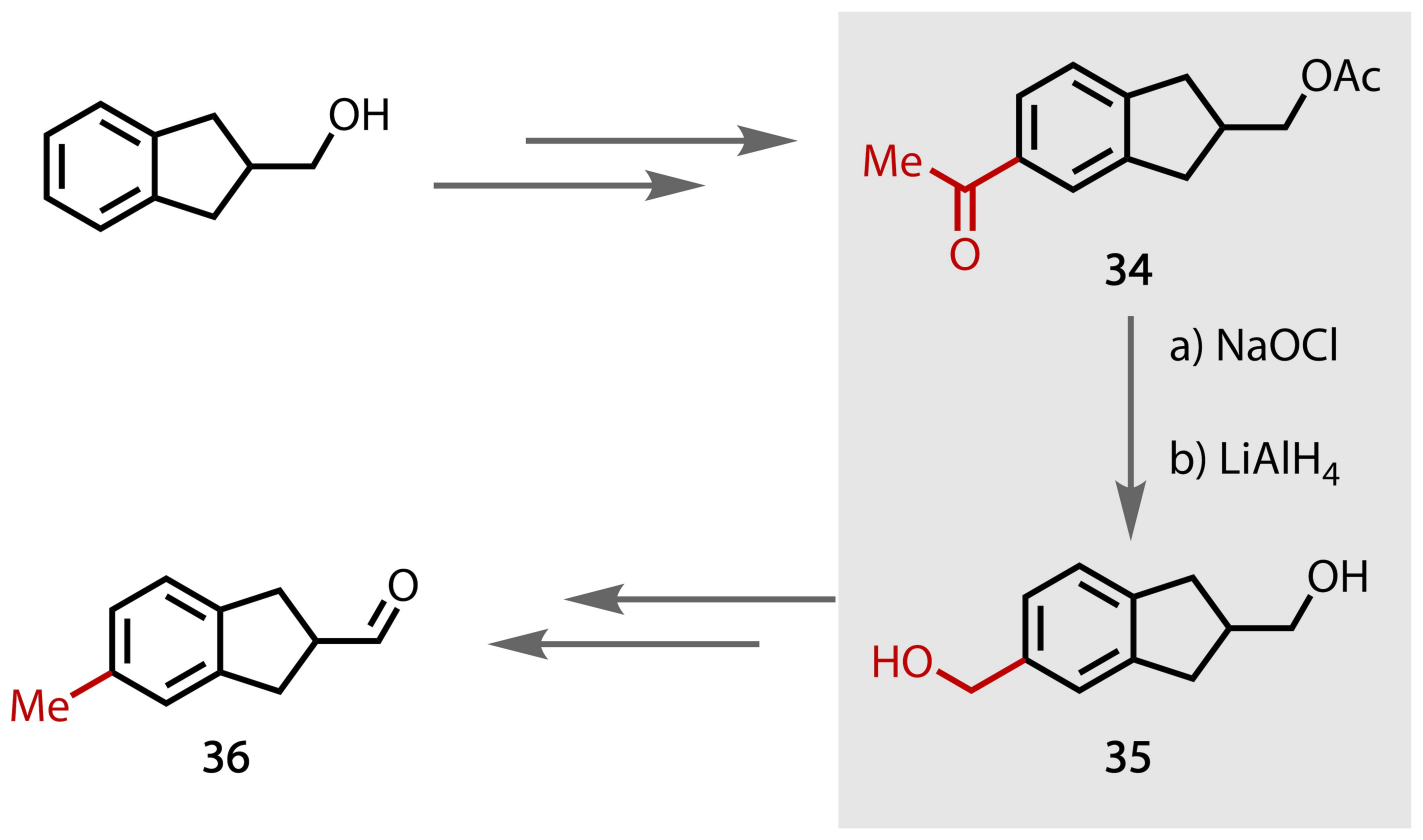
A haloform reaction and reductive decarbonylation yields an alcohol (35) from a methyl ketone (34).

In fine or specialty industrial chemical synthesis the haloform reaction has been used in the synthesis of synthetic fragrances. Winter and co‐workers reported a synthesis of derivatives of substituted indane‐2‐carboxaldehydes (**36**) related to floral‐type odorants in order to assess the effect of modifying the lipophilic component on olfactory properties.^[133]^ The authors utilised the methyl ketone **34** to carboxylic acid transformation paired with an *in situ* reduction to alcohol **35** enroute to their fragrant **36**, Figure 37.

**Figure 38:**
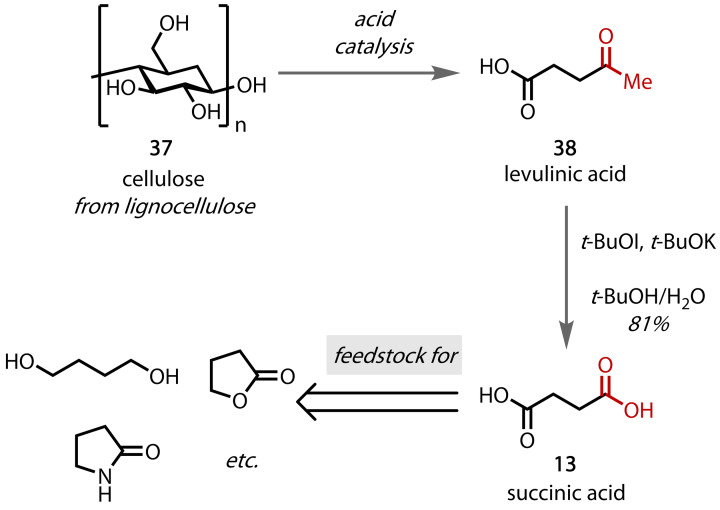
Production of the commodity chemical succinic acid (13) from lignocellulosic biomass (37).

The haloform reaction has shown promise in the industrial production of commodity chemicals via biomass valorisation. Uchiyama and co‐workers demonstrated that succinic acid (**13**), a major four‐carbon chemical feedstock, can be obtained via a haloform reaction with levulinic acid (**38**), which can be produced in a single step from lignocellulose (**37**), Figure 38.^[91]^ Other methods for the conversion of levulinic acid (**38**) to succinic acid (**13**) have considerable drawbacks and, indeed, Uchiyama found that *tert*‐butyl hypoiodite (*t*‐BuOI) as the oxidant, formed *in situ* from iodine and potassium *tert*‐butoxide, enabled the selective synthesis of succinic acid (**13**) in high yield at room temperature. Chromatography‐free, gram‐scale synthesis was demonstrated, as was a one‐pot synthesis from cellulose, suggesting there may be potential for the development of a sustainable, low‐cost process for the valorisation of non‐edible lignocellulosic biomass.

**Figure 39:**
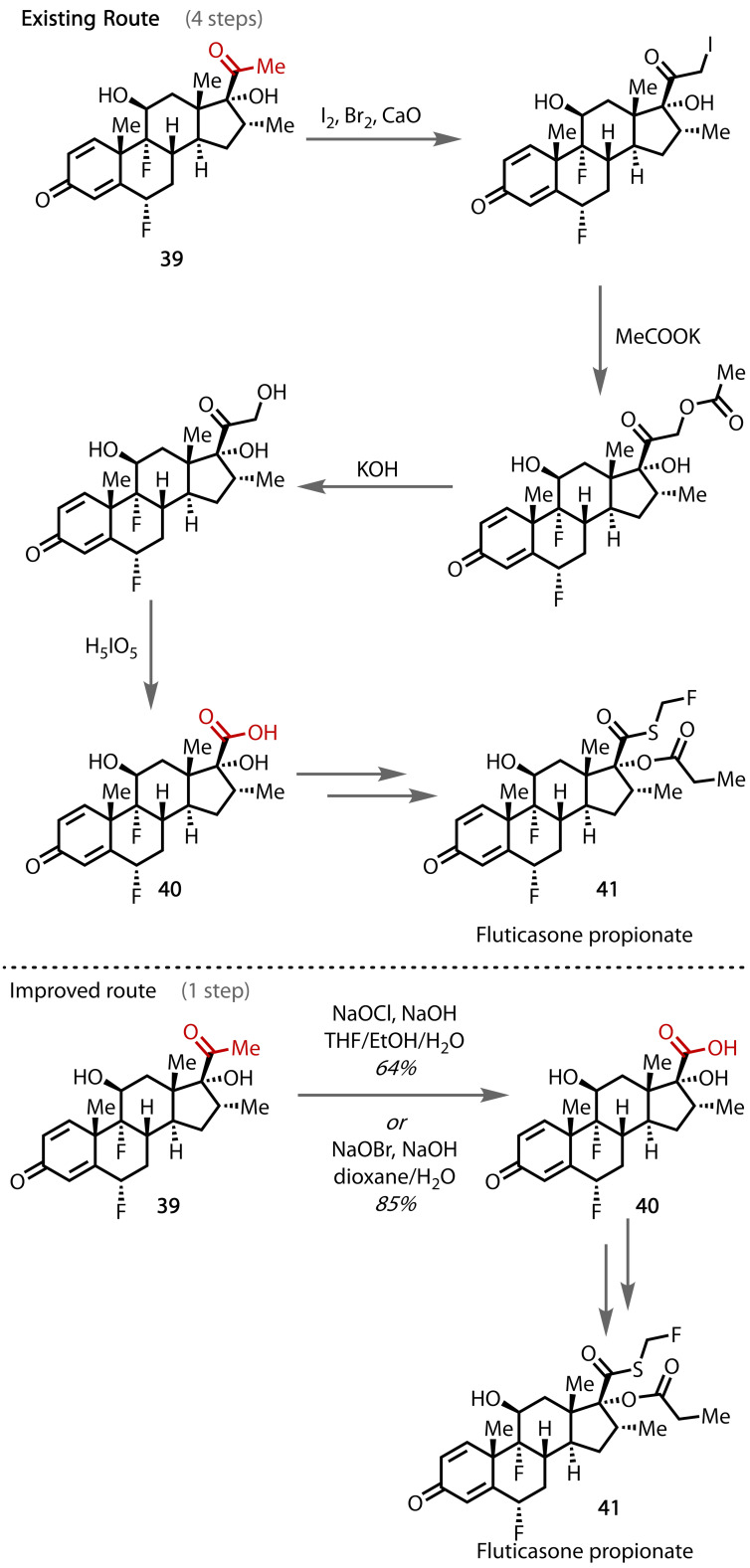
Proposed improvement to the synthetic route to fluticasone propionate (41).

The haloform reaction has been proposed as a means of improving the synthesis of the anti‐inflammatory drug, fluticasone propionate (**41**). Su and co‐workers envisaged that the existing 4‐step conversion of methyl ketone intermediate **39** to carboxylic acid **40** in the route to the active pharmaceutical ingredient (API) could be accomplished in a single step, Figure 39.^[134]^ They showed that the transformation could be achieved with sodium hypochlorite or hypobromite, giving yields of 64% and 85% on 50 g‐ and 100 g‐scales, respectively. This shorter route should enable significant cost savings and an improvement in the total yield of API. Since the reaction was demonstrated on an industrially‐relevant hectogram (100 g) scale and requires only inexpensive, readily‐available reagents, further scale‐up and therefore commercialisation was deemed viable.

**Figure 40:**
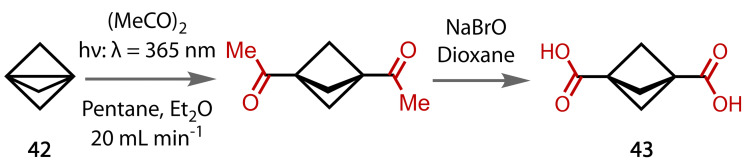
Mykhailiuk's scale‐up of Michls two‐step process to bicyclopentane diacid 43, with the haloform reaction as the second step.

Bicyclo[1.1.1]pentane‐1,3‐dicarboxylic acid (**43**) is a useful intermediate for the formation of a range of important building blocks. Bicyclopentane is a bioisostere of benzene that is increasingly being used in active pharmaceutical agents. Michl and co‐workers took propellane **42** and doubly acetylated it under photochemical conditions to form the diacetyl bicyclopentane, Figure 40.[^135^, ^136^] This compound was subjected to haloform conditions to form the diacid **43** in excellent yield. The 25 g‐scale demonstrated by Michl, was greatly increased by Mykhailiuk and co‐workers who ran the photochemical step in flow on kg scale, and then demonstrated the haloform step on 125 g‐scale.^[137]^ They made 0.5 kg by running the haloform reaction 4 times.

The following examples detail natural products that have been synthesised with the haloform used as a key step in the synthesis. The majority of examples that have used a full haloform reaction directly form the carboxylic acid, with only a small number of ester forming examples and there are no examples of direct amide synthesis. Additionally, there are many examples from total synthesis where a partial haloform reaction, in which a preinstalled trihalomethyl ketone is converted to a carboxylic acid or ester, has been used. Such examples include Heranonapyrrole C,^[47]^ Marinopyrolle B,^[29]^ Jasminine,^[33]^ Malagashanine^[35]^ and Xyloketal A.^[34]^ However, a discussion of these examples is outside the scope of this review.

### 3.1 Smenospondiol

**Figure 41:**
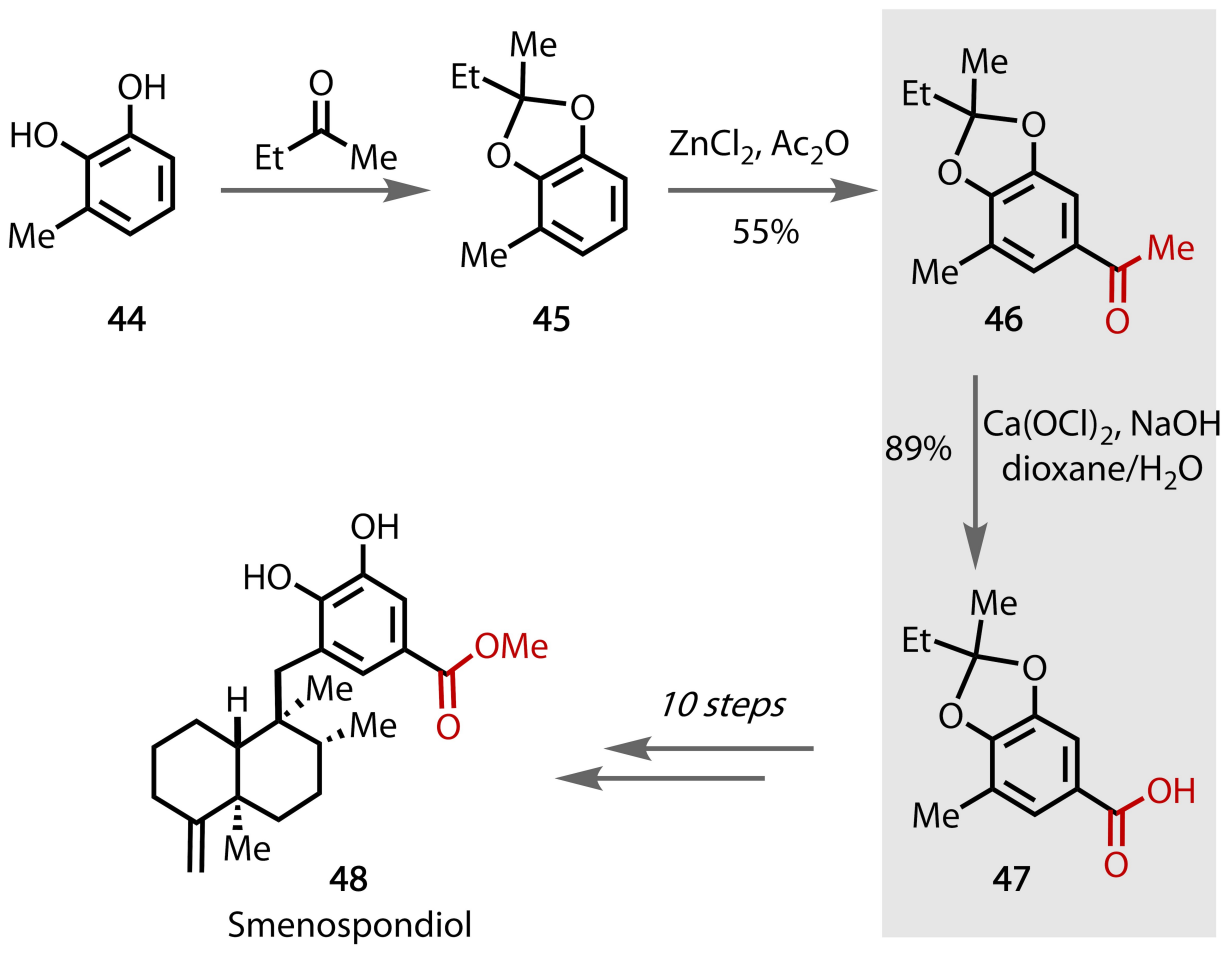
Total 13‐step synthesis of smenospondiol (48).

Smenospondiol **48** has been shown to exhibit antimicrobial, antiviral and hypoxia‐selective growth inhibitory properties,^[138]^ which could find applications in anticancer therapies.^[139]^ Following a racemic synthesis reported by Haruo *et al*.,^[140]^ Sumii *et al*. completed an enantioselective synthesis of smenospondiol (**48**), Figure 41, involving the haloform step early in the synthesis.^[141]^

Starting from commercially available 3‐methylcatechol **44**, the diol was protected as a cyclic ketal to afford **45** and subsequent installation of the methyl ketone moiety via Friedel–Crafts acylation afforded methyl ketone species **46**. Haloform reaction of **46** using Ca(ClO)_2_ and NaOH in a dioxane/water solvent mixture afforded the corresponding benzoic acid **47**. This transformation proceeded in 89% yield and allowed for the overall conversion of the aromatic sp^2^ C−H to a carboxylic acid group in two simple steps using inexpensive and widely available reagents. The carboxylic acid group of **47** was key to the synthesis of the methyl ester found in the target compound, affording smenospondiol **48** after 10 further steps.

### 3.2 (±)‐9‐Isocyanopupukeanane

**Figure 42:**
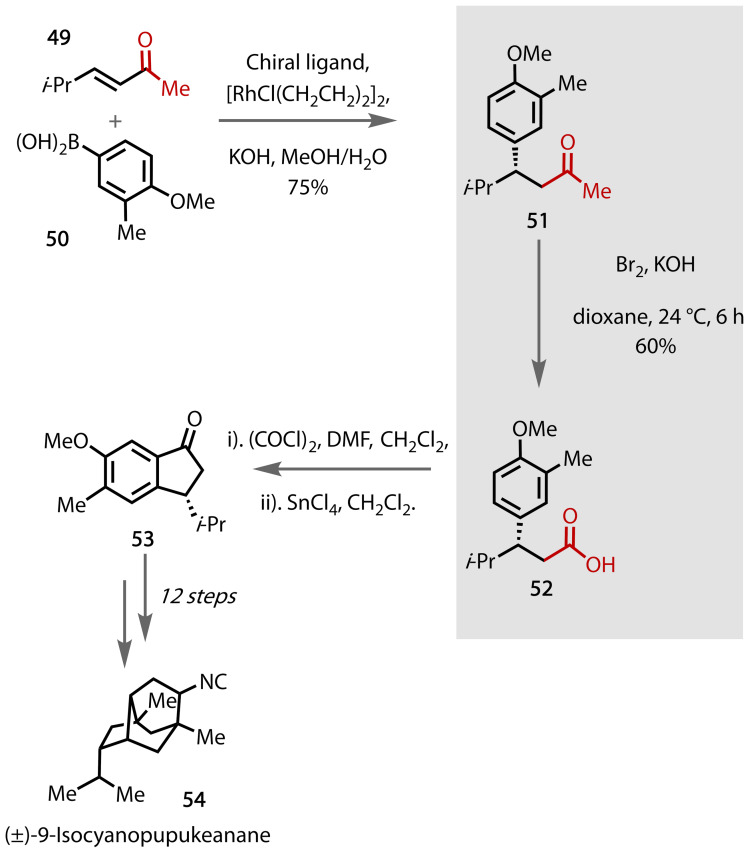
Total synthesis of (±)‐9‐isocyanopupukeanane (54).

The sesquiterpene isocyanide (±)‐9‐isocyanopupukeanane **54** was isolated in 1979,^[142]^ and has gained interest due to the potent antimalarial activity of such isocyanoterpenes.^[143]^ Building on the racemic total synthesis published by Corey in 1979,^[144]^ Corey and Brown published an enantioselective synthesis of key intermediate **53** in 2010 utilising the haloform reaction, Figure 42.^[145]^

Commercially available α,β‐unsaturated ketone **49** provides the key methyl ketone functional group, installed via rhodium‐catalysed conjugate addition of boronic acid **50** to **49**, affording chiral ketone **51**. The methyl ketone was transformed via a haloform reaction to the corresponding carboxylic acid **52** using Br_2_ and KOH in dioxane. In this dialkylketone (**51**), halogenation of the more accessible methyl group was favoured over the internal methylene group. The carboxylic acid was key for the subsequent alkylation, hydrolysis, and methylation steps to afford the key chiral intermediate **53**. The authors propose (±)‐9‐isocyanopupukeanane **54** could subsequently be afforded in 12 steps using Corey's achiral methodology.^[144]^ In this instance, using a haloform reaction enabled the installation of the carboxylic acid from an inexpensive and readily available starting material; the desired carboxylic acid **52** is not readily available.

### 3.3 (±)‐Anthoplalone

**Figure 43:**
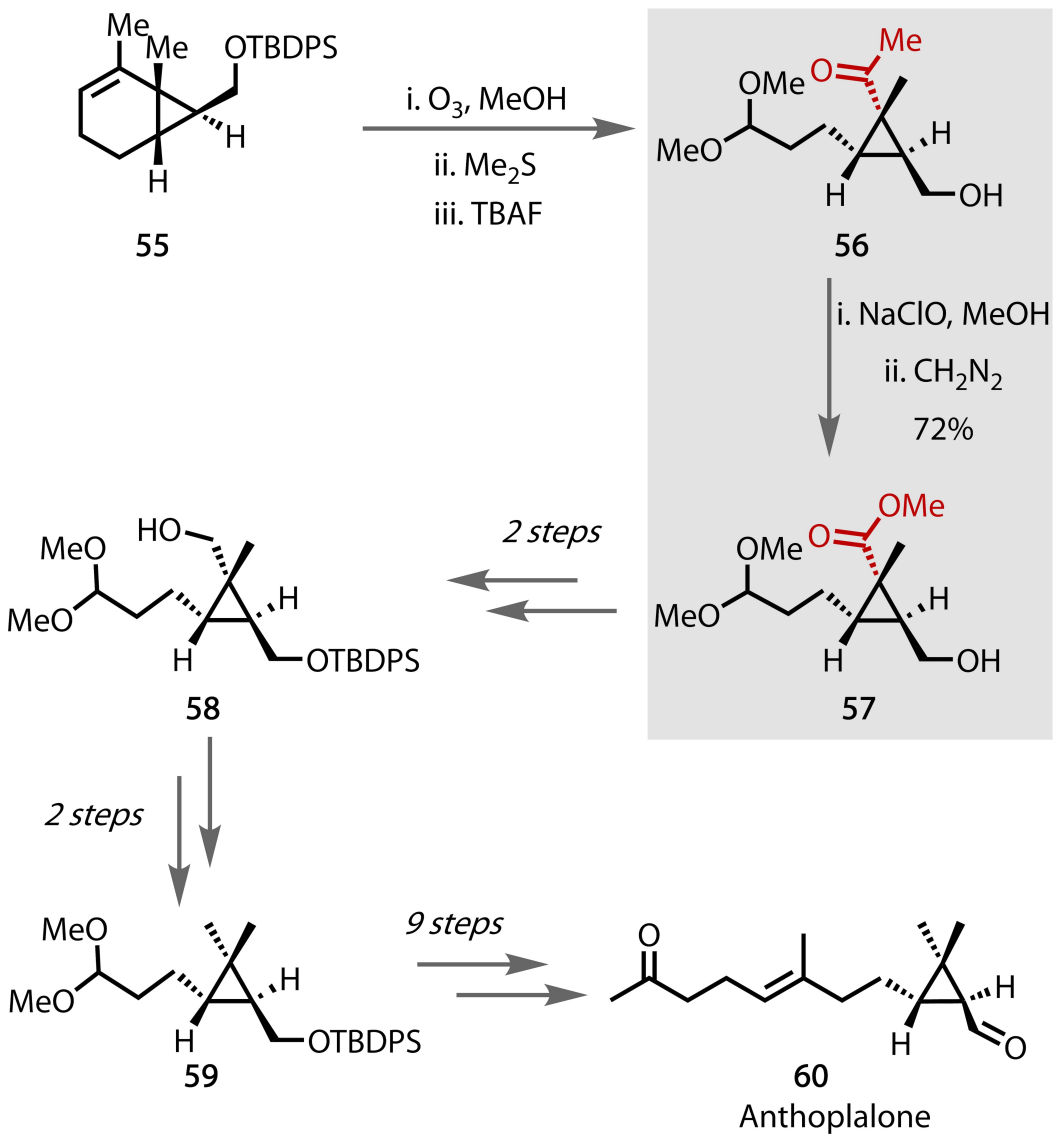
Total synthesis of anthoplalone 60, synthesised by Ihara and coworkers in 1994.

Secosesquiterpene (±)‐anthoplalone **60** was isolated in 1990 and exhibits cytotoxicity against murine melanoma cells.^[146]^ Ihara and co‐workers reported the total synthesis of (±)‐anthoplalone **60**, utilising the haloform reaction to reduce the acetyl side chain length by one carbon, Figure 43.^[147]^ Compound **55** was synthesised in 6 steps from commercially available caprolactone, and the methyl ketone moiety was installed via ozonolysis. Deprotection of the alcohol group afforded **56**, which was subjected to the haloform reaction using NaClO in MeOH to afford the carboxylic acid that gave the corresponding ester **57** upon further reaction with CH_2_N_2_. The alcohol was protected, and the ester was reduced to afford **58**, which, following mesylation and reduction, afforded compound **59** containing the key dimethylcyclopropane moiety. (±)‐Anthoplalone **60** was afforded after 9 subsequent steps.

### 3.4 Heliolactone

**Figure 44:**
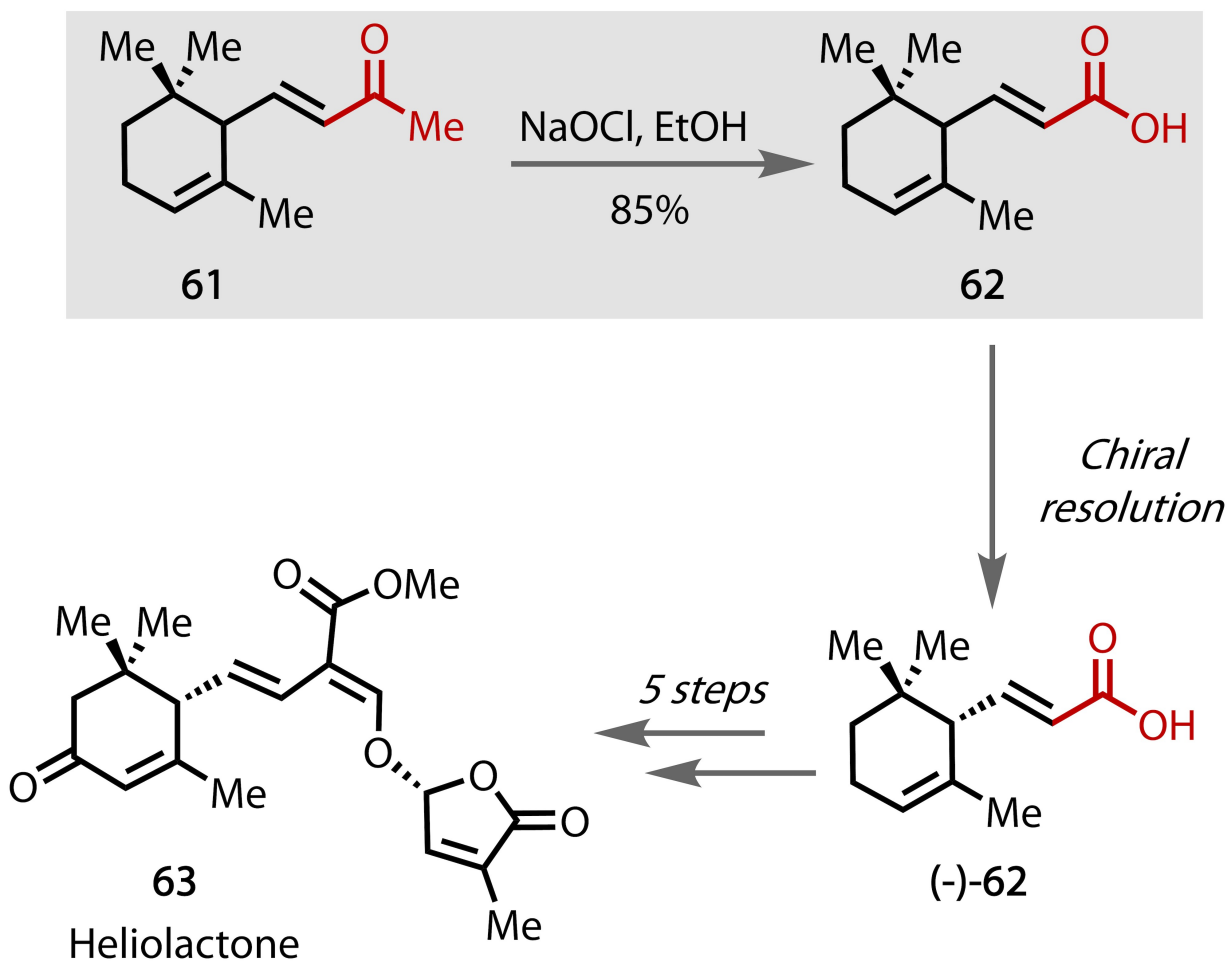
Total synthesis of heliolactone (63).

Heliolactone, a member of the strigolactone family, was initially isolated in 2014 and is a phytohormone that plays a key role in the growth and development of plant roots.^[148]^ An enantioselective synthesis towards chiral heliolactone **63** was described by Woo and McErlean, Figure 44,^[149]^ beginning with a haloform reaction on the commercially available racemic terpene α‐ionone **61**. The corresponding carboxylic acid (**62**) was obtained as a single enantiomer (**(−)‐62**) through chiral resolution, from which heliolactone **63** was obtained in 5 steps.

### 3.5 (±)‐Methyl Jasmonates

**Figure 45:**
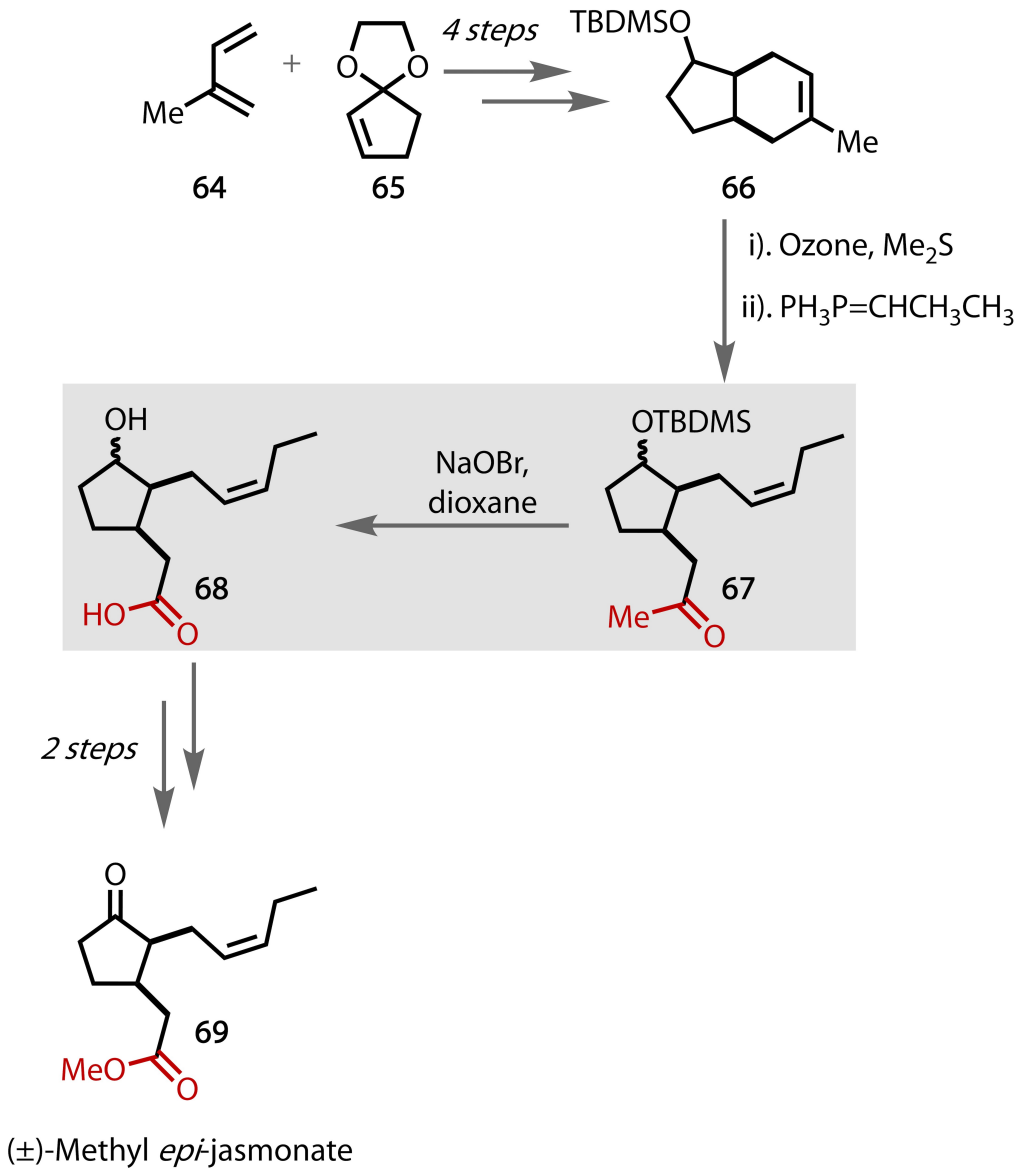
Total synthesis of (±)‐methyl *epi*‐jasmonate (69).

Methyl jasmonates are volatile compounds with broad applications in perfumes, with the *epi*‐isomer possessing the strongest odour.^[150]^ Additionally, the *epi*‐isomers of the methyl jasmonates have also been found to exhibit desirable bioactivities in both plant defence and signal transmission.[^151^, ^152^] Previous routes have employed Diels‐Alder strategies, but afford low yields and mixtures of diastereoisomers, so a modified methodology was required.^[153]^ The total synthesis of (±)‐methyl *epi*‐jasmonate **69** was reported by Hailes and co‐workers utilising a haloform reaction late in the synthesis, Figure 45.^[154]^

Diels‐Alder cyclisation between readily available diene **64** and 1,4‐dioxaspiro[4.4]non‐6‐ene **65**, followed by deprotection of the ketal, reduction of the given ketone and protection of the resultant alcohol afforded intermediate **66**. To install the methyl ketone moiety, **66** was subjected to ozonolysis followed by a reductive workup. Subsequent Wittig reaction afforded species **67**, which underwent a haloform reaction to yield carboxylic acid **68**, with simultaneous deprotection of the alcohol group. The carboxylic acid was methylated, and the alcohol group was oxidised to afford (±)‐methyl *epi*‐jasmonate **69**.

### 3.6 Veratric Acid

**Figure 46:**
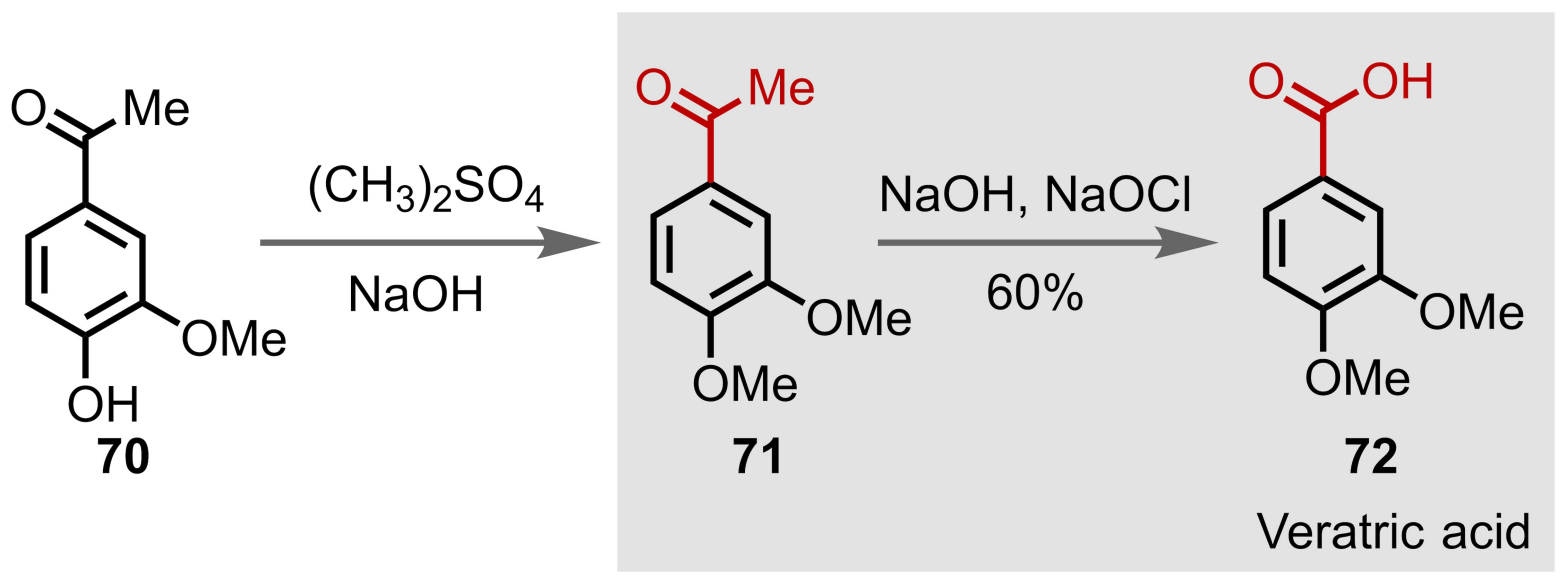
Synthesis of veratric acid (72).

Veratric acid, **72**, is a commonly‐used intermediate in the synthesis of pharmaceuticals, such as meberverin.^[100]^ Bjørsvik and Norman completed a total synthesis of veratric acid **72** from the readily available starting material acetovanillon **70**, which contains a methyl ketone moiety, Figure 46.^[100]^ Methylation of the free hydroxyl group affords **71**, which undergoes a haloform reaction to give the corresponding carboxylic acid group in **72**. Competitive chlorination of the electron‐rich ring affords a minor side product, but its formation could be minimised by monitoring the temperature and concentrations of sodium hypochlorite. This reaction was later demonstrated using flow chemistry.^[155]^

### 3.7 Silphilperfol‐5‐ene

**Figure 47:**
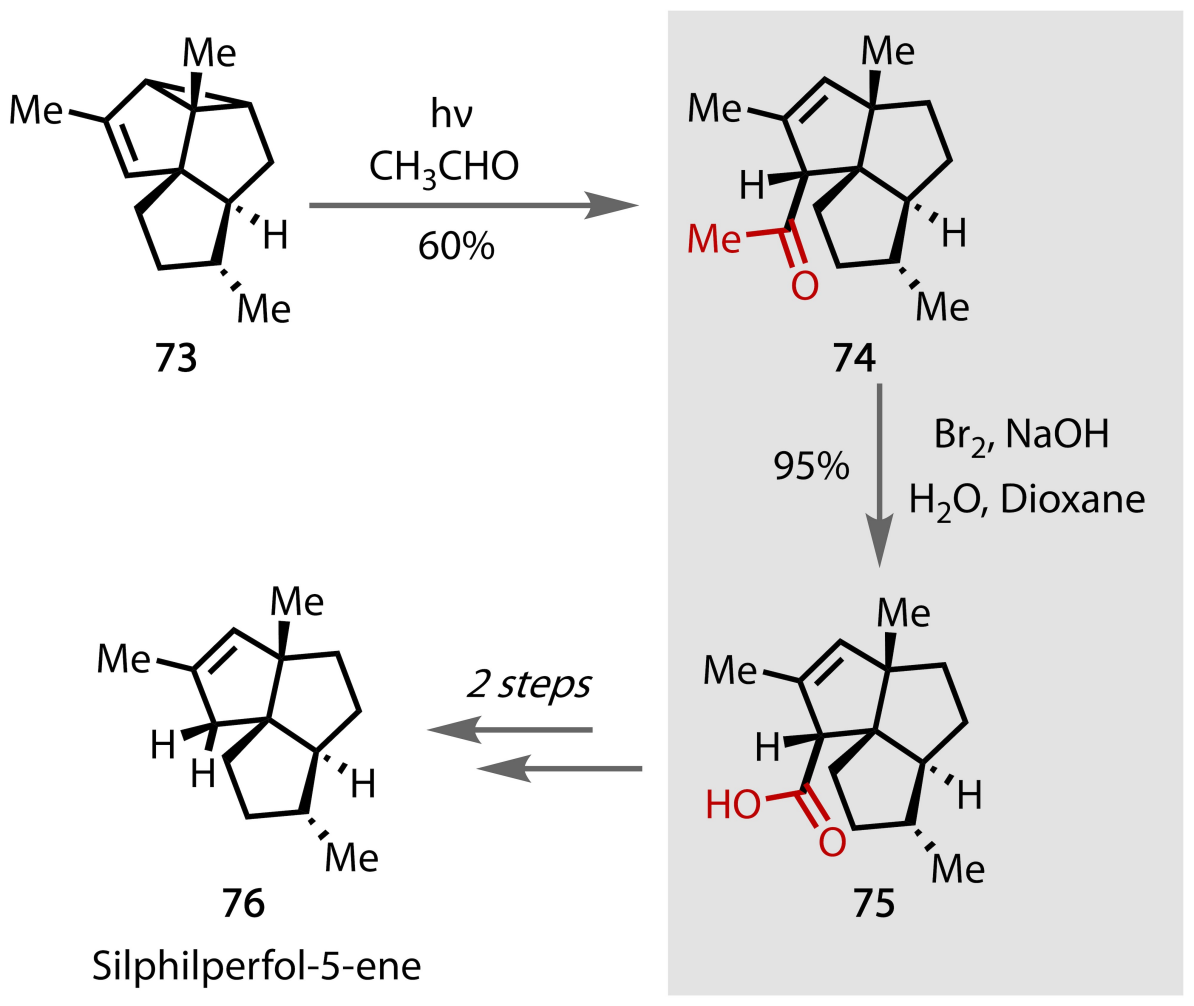
Total synthesis of Silphilperfol‐5‐ene (76).

Silphilperfol‐5‐ene **76** is a sesquiterpene constituent of davana oil, an essential oil that has been tested for antibacterial and wound‐healing properties.^[156]^ Wender and Wasserman completed a total synthesis of **76** from bromo *p*‐xylene and 3‐methyl‐4‐pentenal, Figure 47.^[157]^ Photochemical conditions were used to form **73** through radical cyclisation cascades. Light irradiation of **73** in acetaldehyde afforded the methyl ketone containing species **74**. Subsequent conversion to the carboxylic acid containing compound **75** proceeded via a haloform reaction in 95% yield. The carboxylic acid group was further reduced to the alcohol, which, after deoxygenation, afforded the target compound **76**.

### 3.8 (+)‐(*S*,*S*)‐(*cis‐*6‐Methyltetrahydropyran‐2‐yl)Acetic Acid

**Figure 48:**
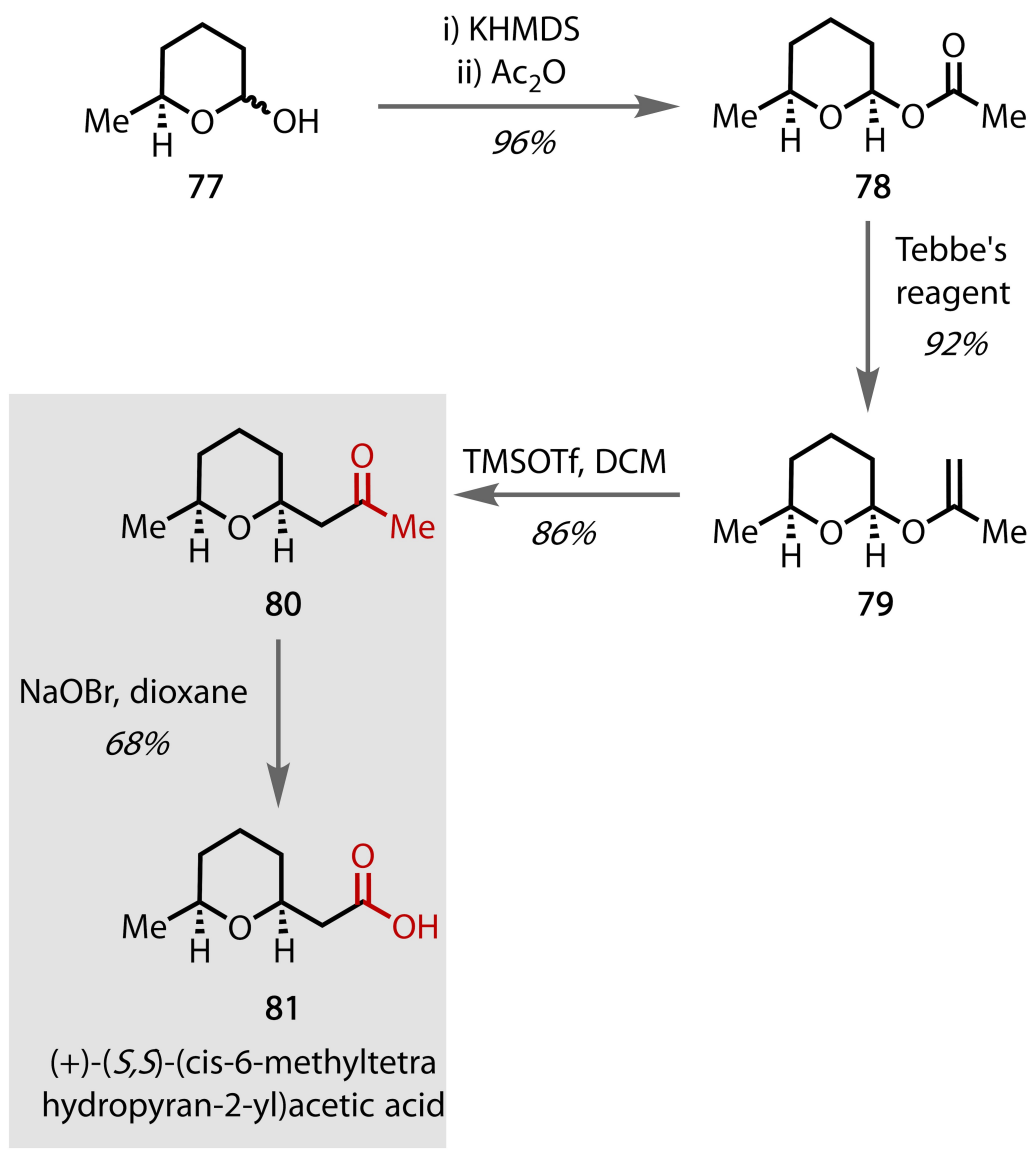
Total synthesis of (+)‐(*S*,*S*)‐(*cis*‐6‐methyltetrahydropyran‐2‐yl)acetic acid (81).

(+)‐(*S,S*)‐(*cis*‐6‐Methyltetrahydropyran‐2‐yl)acetic acid **81** was originally isolated by Maurer and co‐workers in 1979 and has been used in the production of high‐end, animal‐derived fragrances.^[158]^ Asymmetric synthesis of the target compound was achieved by Dixon and co‐workers via a six‐step route utilising the haloform reaction in the final stage, Figure 48.^[159]^ Intermediate **77** was synthesised in two steps from commercially available (−)‐(*S*)‐propylene oxide through copper‐catalysed ring opening with butenyl Grignard and subsequent ozonolysis. Acylation afforded methyl ketone **78**, which upon treatment with Tebbe's reagent, rearranged to enol ether **79**. Lewis acid‐mediated rearrangement with TMSOTf afforded *cis*‐methyl ketone **80**, which was converted to the carboxylic acid via the haloform reaction to afford **81**. Selectivity for enolisation at the terminal protons was achieved using steric control from the cyclohexane ring, which favoured enolisation at the least substituted position to afford the natural product **81**.

### 3.9 Luzopeptins

**Figure 49:**
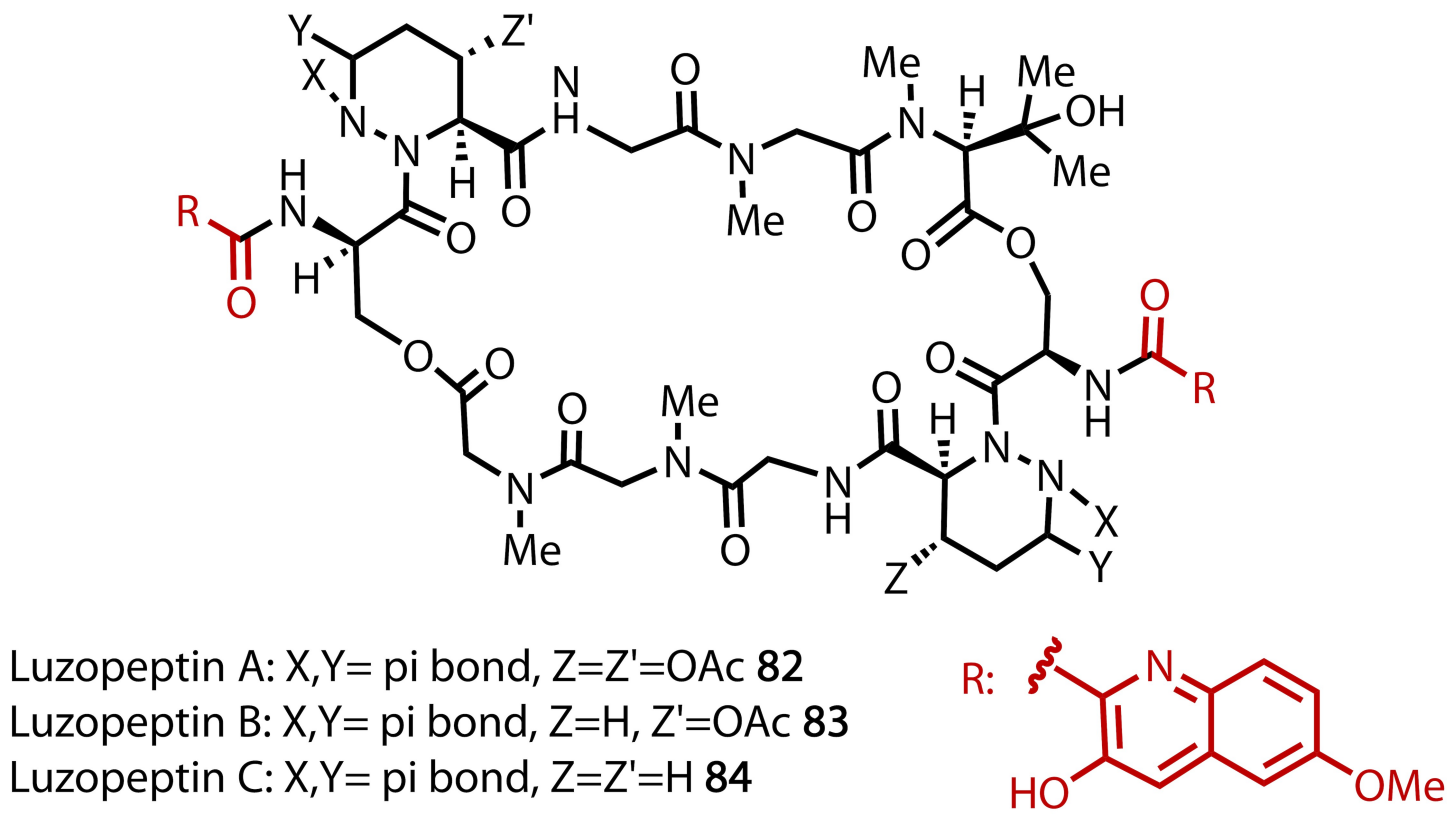
Luzopeptin A−C structures (82–84). Quinaldic acid derived groups in red.

The cytotoxic luzopeptins (**82**–**84**), Figure 49 were discovered by Ohkuma and co‐workers in 1980 and have since been found to exhibit antitumor properties.^[160]^ The complex structure of these peptides are comprised of six main components, including quinaldic acid **88**, a compound whose total synthesis was achieved by Ciufolini and co‐workers in 2005, Figure 50.^[161]^ Quinoline **86** was prepared from commercially available **85** through sequential formation of the acyl chloride and acetyl‐functionalised nitrobenzene using COCl_2_, (MeCO)_2_CH_2_ and NaH. The phenolic alcohol was protected using a benzyl group and through a haloform reaction, the methyl ketone moiety of **86** was transformed into the corresponding carboxylic acid **87**. Subsequent deprotection of the alcohol moiety afforded quinaldic acid **88**. A detailed discussion of the formation of the peptide bonds found in the luzopeptin natural products is available in a report by Cuifolini.^[162]^

**Figure 50:**
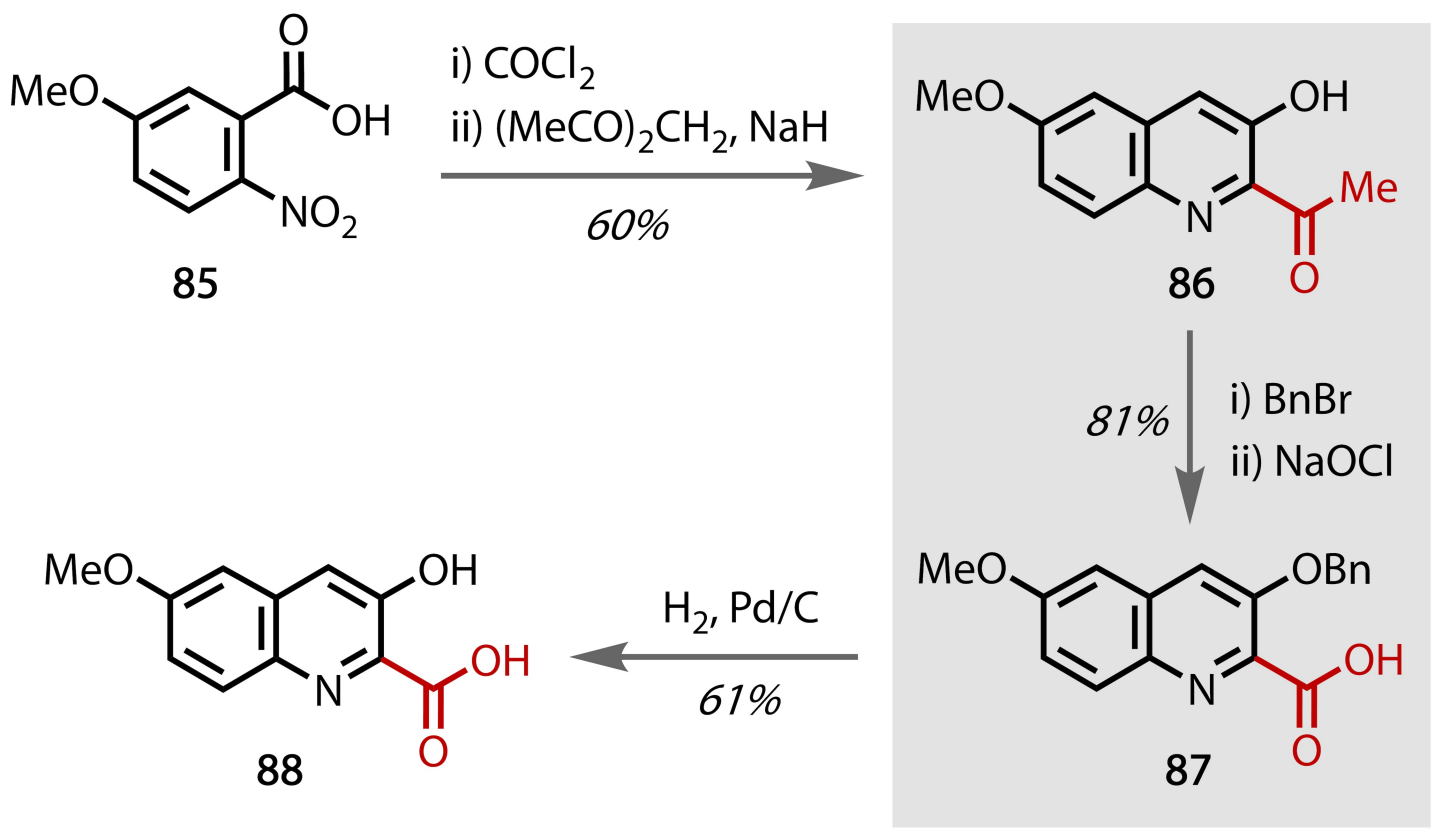
Total synthesis of quinaldic acid (88), a major component of the complex luzopeptin natural products.

### 3.10 Balanol

Balanol **94**, initially isolated from the fungi *Verticillium balanoides* and *Fusarium merismoides*, acts as a protein kinase C (PKC) inhibitor.^[163]^ PKC is involved in cell growth and metabolism, often found in cell inflammation and tumour growth. The synthesis of the balanol intermediate **93** was described by Storm and Anderson in 1999, Figure 51.^[164]^ Through an eleven‐step synthesis starting from **89**, methyl ketone‐containing species **91** was afforded from **90** through bromination, base‐induced HBr elimination and Lemiuex‐Johnson oxidation. This species, containing a methyl ketone moiety, was subjected to a haloform reaction from which the corresponding carboxylic acid **92** was afforded that was hydrolysed to diacid **93**. Warner and co‐workers reported a synthesis of balanol **94** from **93**.^[165]^ The authors extended upon this work using a similar methodology to afford a variety of unsymmetrically‐substituted benzophenones.^[166]^

**Figure 51:**
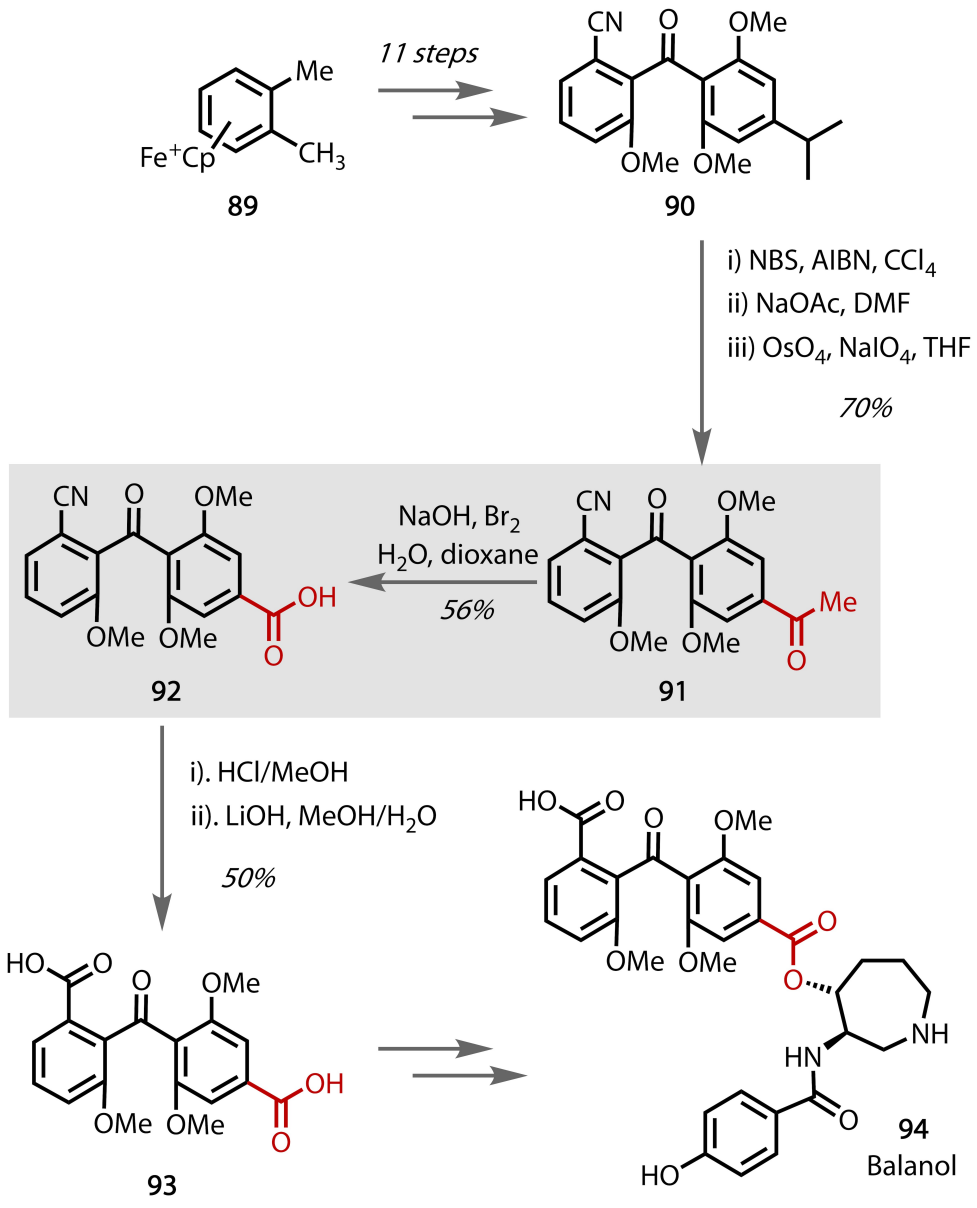
Total synthesis of balanol (94).

### 3.11 Umbrosone

**Figure 52:**
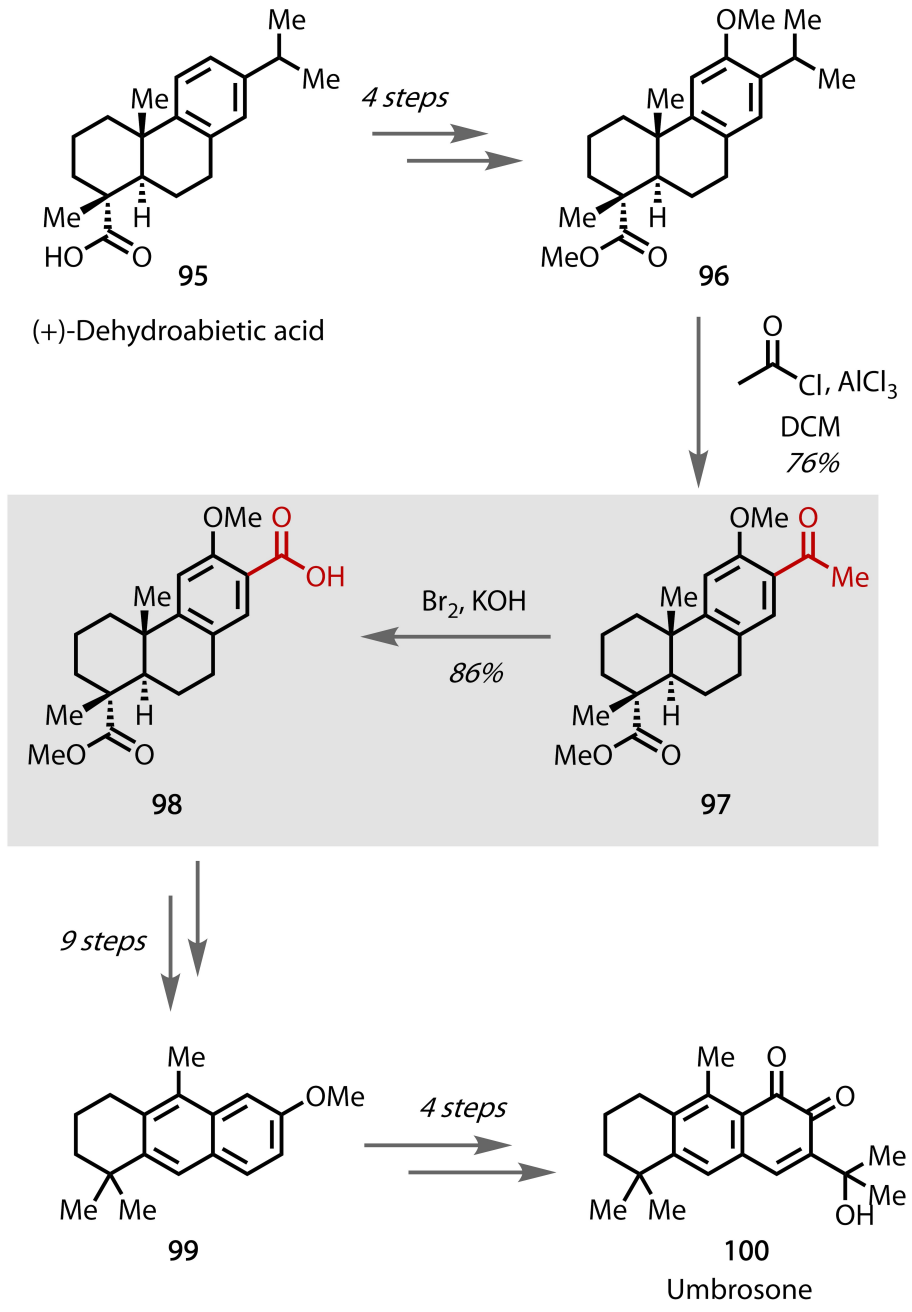
Total synthesis of umbrosone (100).

Umbrosone was first isolated in 1990, and was found to exhibit antimicrobial activity.^[167]^ Using commercially available dehydroabeietic acid **95**, **96** was afforded in 4 steps, Figure 52.^[168]^ The methyl ketone was installed through an unusual dealkylative Friedel‐Crafts acylation of dehydroabietate **97**.^[169]^ A haloform reaction using bromine and KOH transformed **97** to acid **98** in excellent yield. Intermediate **99** was accessed from a further 9 steps,^[170]^ from which the natural product umbrosone **100** can be accessed via previously published work in 4 further steps.^[171]^

### 3.12 (+)‐Upial

(+)‐Upial **105** is a sesquiterpenoid that contains a bicyclo[3.3.1]nonane skeleton.^[172]^ Taschner achieved the first total synthesis of (+)‐upial in 1985,Figure 53.^[173]^ The synthesis began with the readily available terpenoid carvone **101**, and through reductive alkylation, hydrolysis, lactonisation and Swern oxidations, **101** was converted to alkene **102**. Through a further Lemieux‐Johnson oxidation, a methyl ketone moiety was formed in **103**, which underwent the haloform reaction. In this case, the typical carboxylic acid transformation described in previous examples was not observed, but ester product **104** was afforded. The methanol solvent was able to intercept the trihalomethyl ketone to directly form ester **104**. In this case, conversion from the olefin **101** to ester **104** via the haloform reaction was more efficient that other similar routes, such as the McGuirk synthesis.^[174]^

**Figure 53:**
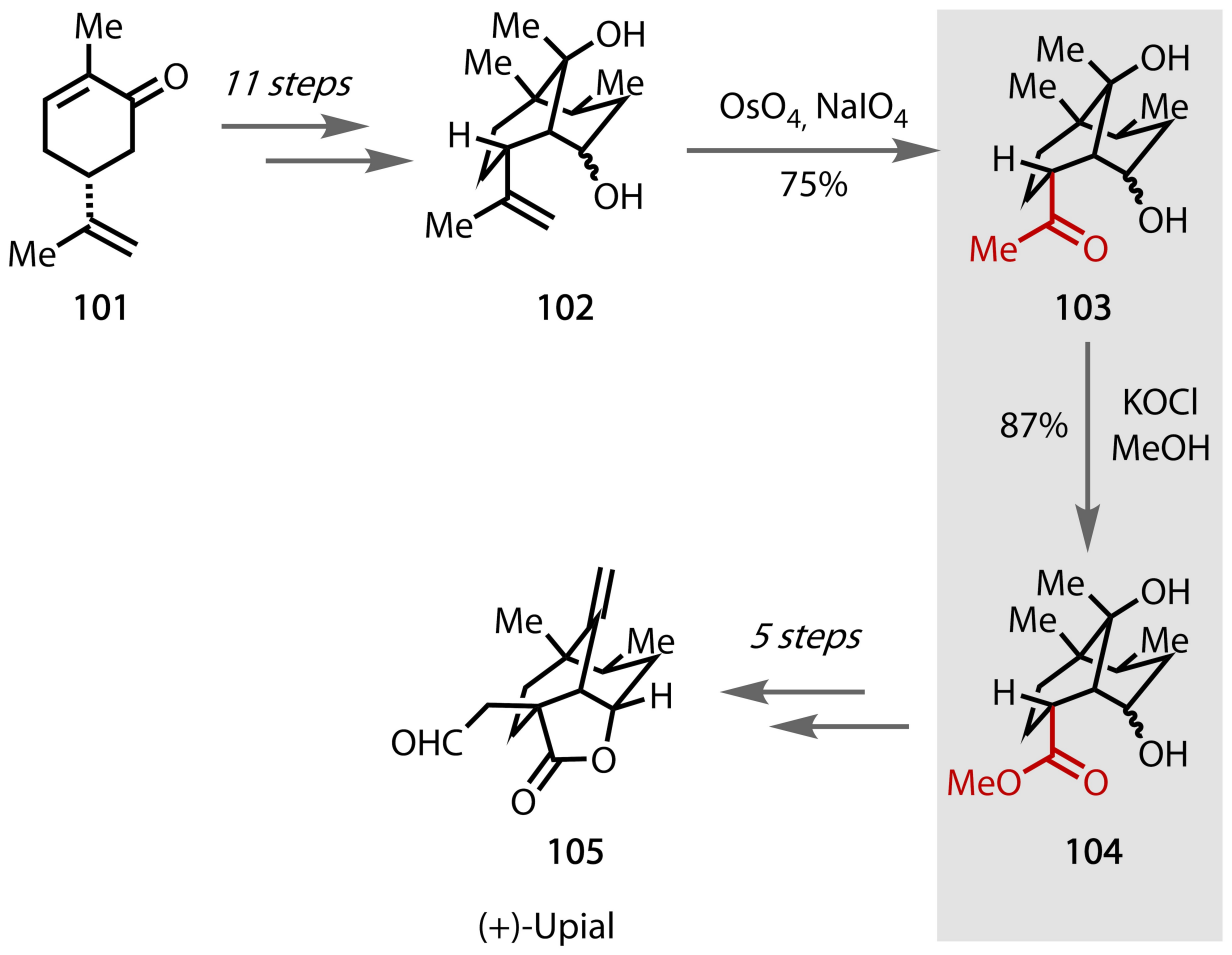
Total synthesis of (+)‐upial (105).

### 3.13 Platensimycin

**Figure 54:**
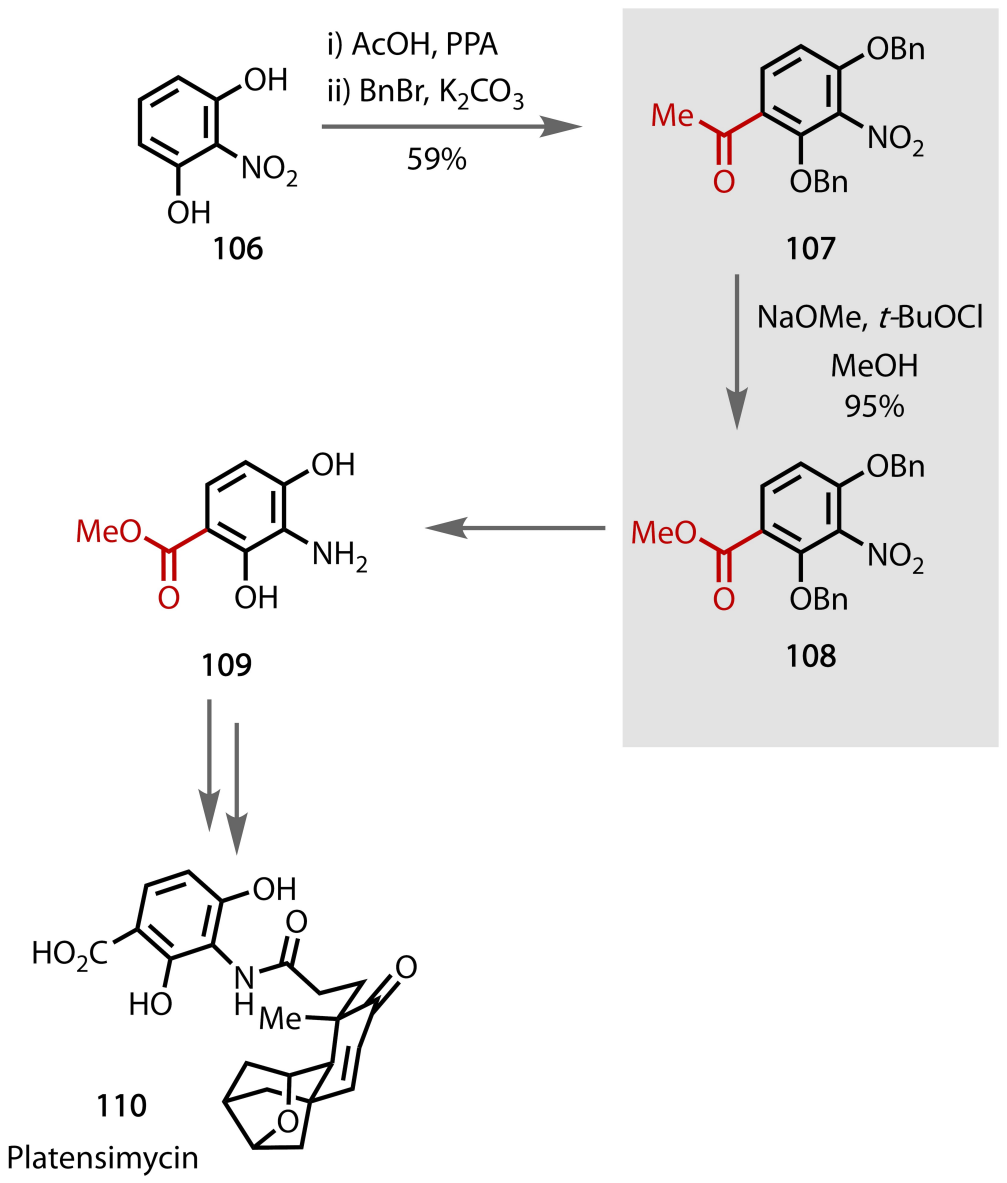
Total synthesis of Platensimycin (110).

Isolated from a strain of *Streptomyces platensis* by researchers at Merck, platensimycin **110** is a potent antibacterial compound, which has shown success in eradicating *staphylococcus aureus* infections in mice.[^175^, ^176^] Eey and Lear reported two routes to form platensimycin **110**. A bismuth‐catalysed route reported in 2010^[177]^ was followed by a route that proceeds via oxocarbenium and iminium intermediates in 2014, the latter of which used the haloform reaction as a key step in the synthesis of intermediate **109**, Figure 54.^[178]^ Starting from 2‐nitroresorcinol (**106**), the methyl ketone moiety was installed through Friedel‐Crafts acylation, and subsequent benzyl protection of the alcohol groups afforded **107**. A haloform reaction using NaOMe, *t*‐BuOCl and MeOH converted the methyl ketone to the methyl ketone in 95 % yield. The protected bisphenol **108** afforded the desired intermediate **109** after benzyl deprotection. Conversion of intermediate **109** to platensimycin **110** was achieved through a subsequent convergent synthesis.^[178]^

### 3.14 Caulersin

Caulersin **116** is a bis‐indole isolated from the marine algae *Caulerpa serrulate*, found in the Xisha Islands of the South China Sea.^[179]^ Various species isolated from this algae have been found to exhibit biological activities, such as antitumor properties and PKC inhibition.^[180]^ Fresneda and co‐workers used the haloform reaction in the first total synthesis of this complex natural product in 1999, Figure 55.^[180]^ Using *N*‐methoxymethyl‐3‐acetyl‐2‐chloroindole (**111**), prepared from 3‐acetyl‐2‐chloroindole and methoxymethylchloride, species **112** was synthesised. The methyl ketone was installed through Lewis‐acid catalysed Michael addition of **112** with methylvinyl ketone, followed by subsequent indole synthesis to afford **113**. Intramolecular nucleophilic substitution afforded the seven‐membered carbocyclic ring, which was subsequently dehydrogenated with DDQ to afford **114**. Conversion of the methyl ketone to the methyl ester using the haloform reaction with KOCl in methanol afforded **115**. A final MOM‐deprotection afforded caulersin **116** in 85 % yield.

**Figure 55:**
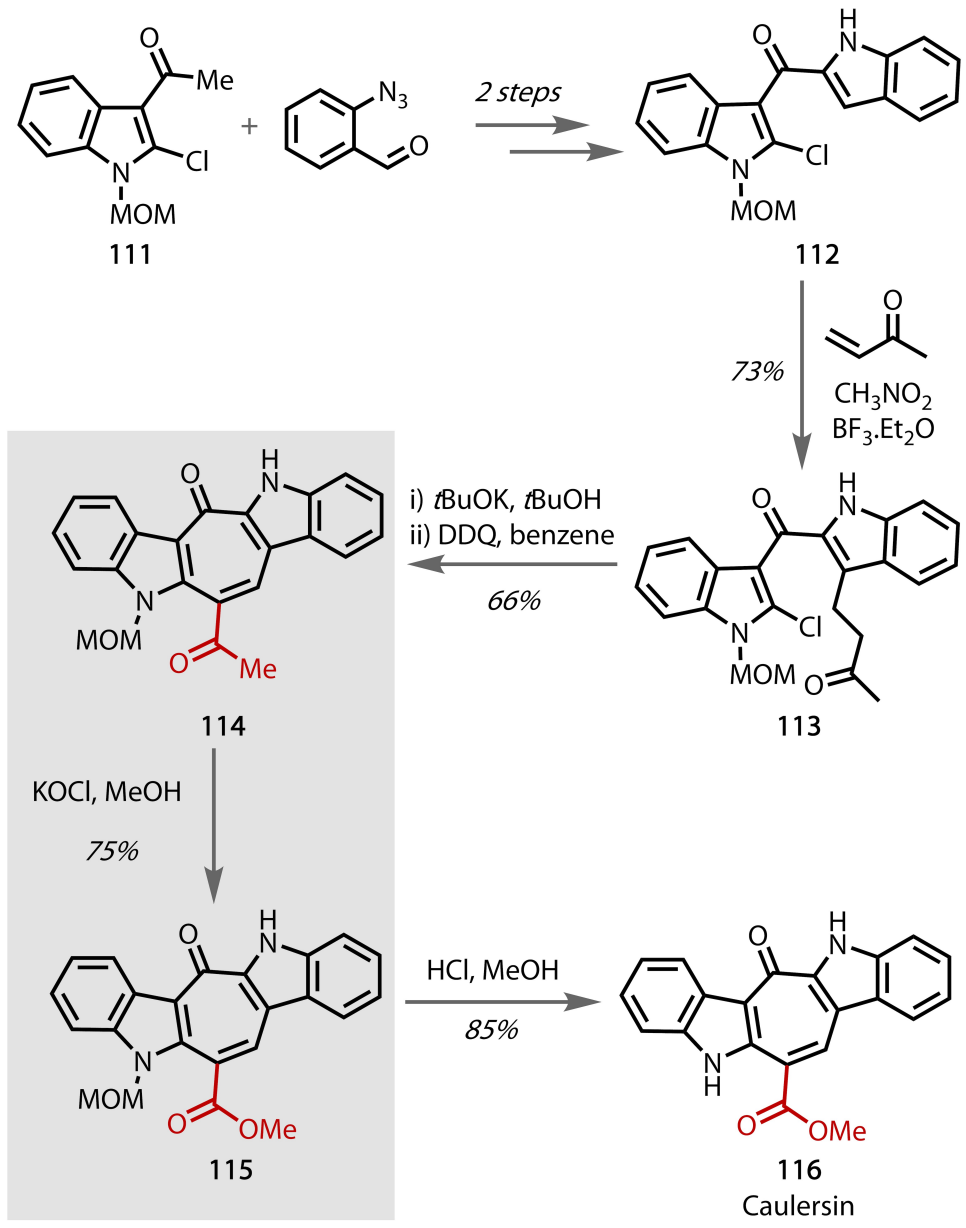
Total synthesis of caulersin (116).

## 4 Conclusions

With over 200 years of history, the haloform reaction is one of the oldest known synthetic organic reactions. The transformation of methyl ketones to carboxylic acids, esters and amides via the exhaustive halogenation of the methyl group under mild conditions enables efficient access to these important functional groups. Having not been reviewed since the 1930s, this review has discussed historical aspects, and the practical and mechanistic advancements of the methodology. A discussion of examples, where the haloform reaction has been applied in the synthesis of complex molecules and natural products, demonstrates the transformation can still be of contemporary value to the synthesis of compounds that can be applied in a variety of useful contexts. Future developments may focus on developing a catalytic approach, finding differently substituted methyl groups that can serve as masked leaving groups to be activated towards C−C bond cleavage following exhaustive halogenation, developing the amidation reaction with more complex amines, and using other nucleophiles to enable new transformations.

## Conflict of Interests

The authors declare no conflict of interest.

5

## Biographical Information

*Albert C. Rowett studied at the University of Bristol (MSci, 2018). He then joined the EPSRC Centre for Doctoral Training (CDT) in Catalysis (University of Bath, University of Bristol and Cardiff University), returning to Bristol to complete his PhD research under the supervision of Dr Alastair Lennox. His research focused on (electro)halogenation reactions and, in particular, the development of a novel haloform coupling methodology*.

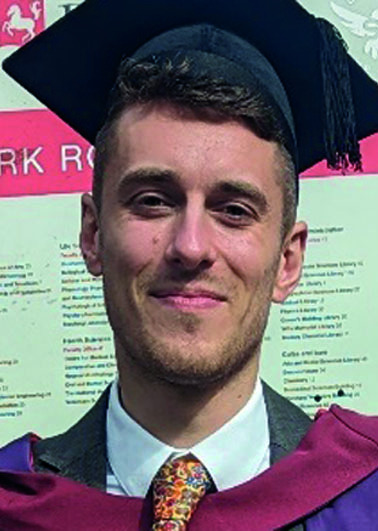

## Biographical Information

*David M. Heard studied at the University of Sheffield (MChem, 2014) and completed a PhD in Chemical Synthesis at the University of Bristol (2019), researching the structural elucidation and total synthesis of maleidride natural products with Prof. Chris Willis. David completed postdoctoral studies with Dr Alastair Lennox (University of Bristol) on electrosynthetic methods and reactor technology, and with Prof. Dennis Hall (University of Alberta) investigating photoredox catalysis in organoboron chemistry. David now works in process development in the pharmaceutical industry*.

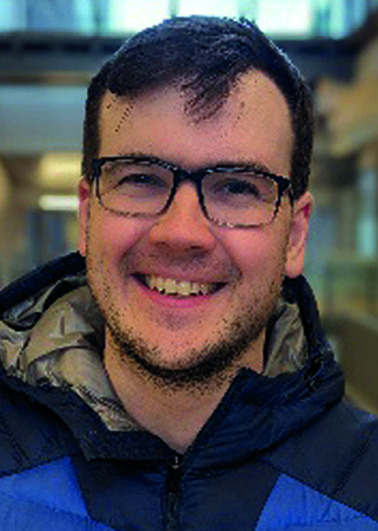

## Biographical Information

*Priya Koria earned her master's degree in Chemistry from the University of Bristol in 2021. She now works in at Unilever within the Global Dishwash Team in Liverpool. Specialising in retail and professional categories, she focuses on developing innovative and sustainable products for global markets. Her expertise drives the creation of cutting‐edge solutions, enhancing Unilever's portfolio and commitment to sustainability*.

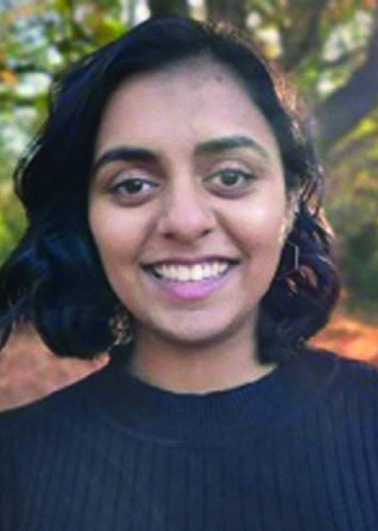

## Biographical Information

*Alice C. Dean received her PhD in 2024 from the Technology Enhanced Chemical Synthesis Centre for Doctoral Training at the University of Bristol working under the supervision of Dr Alastair Lennox. She worked on hypervalent iodine mediated fluorination reactions. Alice now works in medical writing*.

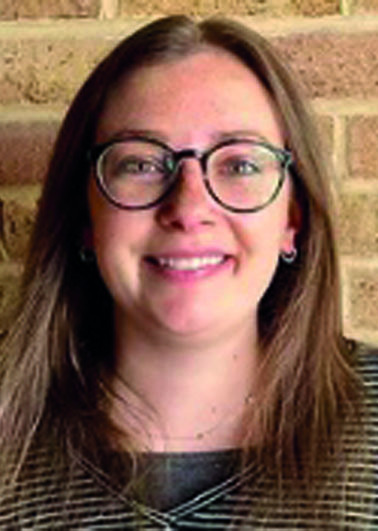

## Biographical Information

*Stephen G. Sweeting received his MSci Chemistry degree from the University of Bristol in 2021. He is now pursuing his PhD under the supervision of Dr Alastair Lennox also at the University of Bristol. His main area of study focuses on understanding fluorination reactions involving hydrogen fluoride complexes, as well as developing new electrochemical methodologies to achieve selective fluorinations*.

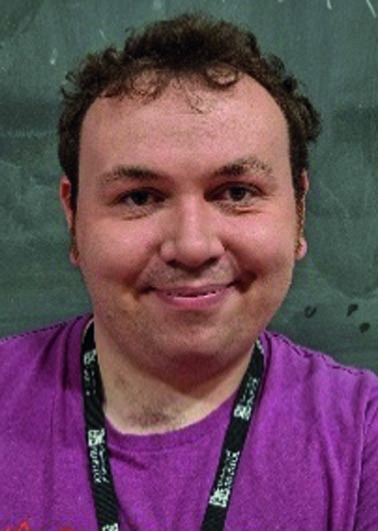

## Biographical Information

*Alastair J. J. Lennox attained his PhD from the University of Bristol (Prof Guy Lloyd‐Jones) and did postdoc studies in Rostock, Germany (Prof Matthias Beller) as an Alexander von Humboldt Fellows and at the University of Wisconsin, Madison (Prof Shannon Stahl). In 2018, Alastair returned to the University of Bristol as a Royal Society University Research Fellow to start his independent research programme. He was promoted to Associate Professor of Chemistry in 2022. His group are interested in the development of novel synthetic organic methods with sustainability and mechanism as themes that strongly underpin their approach to this. Specific interests include the exploration of electrochemistry as a tool for performing selective redox transformations, and also in the development of fluorination reactions and fluorinated building blocks*.

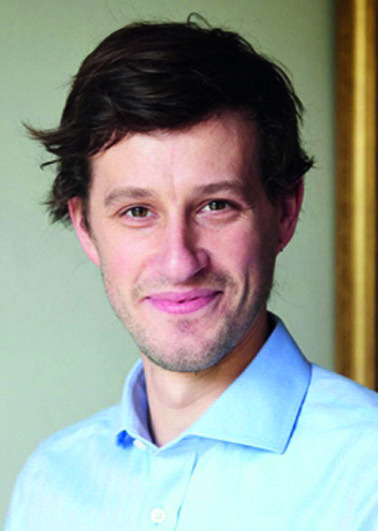

## Data Availability

Data sharing is not applicable to this article as no new data were created or analyzed in this study.
